# Integrative Transcriptomic Analysis and Functional Validation Implicate GPT2 in Glycolytic Regulation in Polycystic Ovary Syndrome

**DOI:** 10.1002/fsn3.72326

**Published:** 2026-09-06

**Authors:** Meili Xi, Rongkui Luo, Jiarong Zhang, Weihong Huang, Li Ma, Yunyan Sun, Aimin Ren

**Affiliations:** ^1^ Obstetrics and Gynecology Zhongshan Hospital, Fudan University Shanghai China; ^2^ Department of Obstetrics and Gynecology, Shanghai Medical College Fudan University Shanghai China; ^3^ Department of Pathology Zhongshan Hospital, Fudan University Shanghai China

**Keywords:** energy metabolism, glycolysis, GPT2, polycystic ovary syndrome

## Abstract

Polycystic ovary syndrome (PCOS) is a heterogeneous endocrine‐metabolic disorder characterized by profound disturbances in energy metabolism, yet the molecular mechanisms underlying glycolytic dysfunction remain incompletely understood. Given the essential role of glycolysis in ovarian function and endocrine homeostasis, this study aimed to systematically characterize glycolysis‐associated molecular alterations in PCOS and identify key metabolic regulators involved in disease pathogenesis. Transcriptomic datasets GSE34526 and GSE6798 were integrated to identify glycolysis‐related differentially expressed genes (GRDEGs). Functional enrichment, immune infiltration, and regulatory network analyzes were performed to characterize the biological features associated with glycolytic dysregulation. Machine learning algorithms, including support vector machine, random forest, logistic regression, and LASSO regression, were applied to prioritize key glycolysis‐associated regulators for downstream biological characterization. The functional role of GPT2 was further examined in KGN granulosa cells under PCOS‐like conditions. Twelve GRDEGs were consistently dysregulated in PCOS and were predominantly enriched in glycolytic metabolism, ATP generation, and transcriptional regulatory processes. Integrative machine learning analyzes prioritized four key glycolysis‐related genes (AMPD3, C5AR1, MLXIPL, and PDLIM7) associated with glycolytic remodeling in PCOS. Immune infiltration analyzes further revealed coordinated metabolic and immune remodeling, while regulatory network analyzes highlighted extensive interactions between hub genes and miRNA‐, transcription factor‐, and RNA‐binding protein‐mediated regulatory networks. Functional experiments demonstrated that GPT2 knockdown impaired glycolytic activity, reduced ATP production and aromatase activity, disrupted steroid hormone homeostasis, and exacerbated metabolic dysfunction in granulosa cells, whereas pharmacological activation of glycolysis partially reversed these alterations. Our findings provide a comprehensive characterization of glycolytic dysregulation in PCOS and identify GPT2 as a potential metabolic regulator linking altered energy metabolism to ovarian dysfunction. These findings provide a molecular framework for future studies investigating metabolism‐ and nutrition‐based intervention strategies in PCOS.

## Graphical abstract

Integrating GEO transcriptomic datasets, machine‐learning algorithms, GSVA, multilayer regulatory network and immune infiltration analyses, this study revealed metabolic‐immune disturbance and aberrant ovarian steroidogenesis in polycystic ovary syndrome (PCOS). Functional experiments in KGN granulosa cells demonstrated that GPT2 knockdown repressed key glycolytic enzymes, reduced ATP and lactate production, and down‐regulated CYP19A1, leading to testosterone accumulation and estradiol deficiency. The glycolysis agonist DPI could partially rescue these injuries induced by GPT2 depletion. This study indicates that GPT2 acts as a critical molecule mediating glycolytic metabolism and steroid hormone homeostasis in granulosa cells.

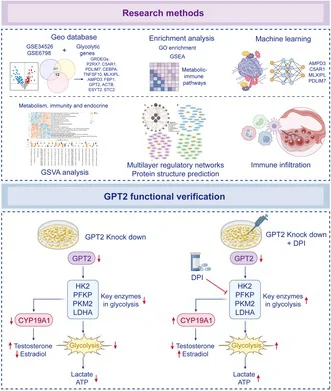

**Abbreviations**
GRDEGs: Glycolysis‐related differential expressed genes
GRG: Glycolysis Related Genes
PCOS: Polycystic Ovary Syndrome
RBP: RNA‐Binding Protein

## 1 Introduction

Polycystic ovary syndrome (PCOS) is a common endocrine and metabolic disorder in women of reproductive age, characterized by marked clinical heterogeneity and long‐term health risks (Azziz et al. 2016; Fauser et al. 2012). In addition to reproductive manifestations including menstrual irregularities and infertility, patients with PCOS are often complicated by insulin resistance, Type 2 diabetes mellitus, and increased cardiovascular metabolic risks, thus making it a major public health concern that impacts women's health across the entire life cycle (Jacobs and Conway 1999; Ganapathy‐Kanniappan and Geschwind 2013). Despite the relatively well‐established clinical diagnostic criteria, the molecular mechanisms underlying disease pathogenesis and phenotypic heterogeneity remain to be comprehensively elucidated.

Energy metabolism reprogramming is recognized as a critical pathological basis of polycystic ovary syndrome (PCOS) (Yan et al. 2024). Among the metabolic pathways, glycolysis plays a pivotal role in energy supply, steroid hormone synthesis, and maintenance of the local immune microenvironment in ovarian cells (Wang and Li 2023; Wang et al. 2025). In particular, ovarian granulosa cells are highly dependent on glucose metabolism to support follicle development and estradiol production, and disruptions in this metabolic axis have been increasingly linked to PCOS pathophysiology (Yan et al. 2025; Zhang et al. 2023). Emerging evidence suggests that the aberrant expression of glycolysis‐related genes may contribute to follicular developmental disorders and hormonal imbalance (Zhu et al. 2025; Zhang et al. 2023). However, most existing studies have focused on individual molecules or local signaling pathways, lacking a holistic transcriptome‐level analysis, and the potential value of these genes in disease classification and clinical diagnosis remains unclear (Wu et al. 2022; Yu et al. 2025). Therefore, systematic dissection of the molecular characteristics underlying glycolytic dysfunction in PCOS is of great significance for deepening the understanding of its pathogenic mechanisms.

From a nutritional perspective, dietary carbohydrate quality and quantity are major determinants of postprandial glycemic response and insulin secretion. High‐glycemic‐load diets have been consistently associated with an increased risk of PCOS and aggravated metabolic phenotypes, likely through exacerbating hyperinsulinemia and peripheral insulin resistance (Zhang et al. 2025; Cutler et al. 2019). Insulin resistance, in turn, impairs ovarian glucose uptake and utilization by disrupting the insulin/PI3K/AKT signaling cascade, which ultimately compromises glycolytic flux in granulosa cells and alters the availability of metabolic intermediates required for steroidogenesis (Rice et al. 2005; Diamanti‐Kandarakis et al. 2016). Furthermore, recent studies have indicated that excessive glucose flux via the polyol or hexosamine pathways, rather than enhanced glycolysis, may contribute to mitochondrial dysfunction and oxidative stress in PCOS ovaries, further linking dietary carbohydrate overload to reproductive metabolic disturbances (Yan et al. 2024; Ramírez‐Martínez et al. 2024). Despite these recognized associations, systematic investigations that bridge dietary glycemic indices, peripheral insulin resistance, and ovarian glycolytic transcriptional signatures remain scarce, and whether specific glycolysis‐related genes can serve as robust diagnostic or nutritional intervention targets has yet to be established.

Therefore, we hypothesize that PCOS‐associated glycolytic dysregulation is not merely an intrinsic ovarian defect, but is closely intertwined with systemic metabolic states influenced by dietary carbohydrate exposure, and that glycolysis‐related molecular signatures may collectively define distinct pathophysiological subtypes. In this study, we performed an integrated transcriptomic analysis to systematically characterize glycolysis‐related molecular alterations in PCOS and identify key metabolic regulators associated with disease pathogenesis. We combined bioinformatics analyzes with machine learning approaches to evaluate the biological and clinical relevance of glycolysis‐related genes, followed by experimental validation of a representative candidate in granulosa cells. Our findings provide new insights into glycolytic dysregulation in PCOS and establish a molecular framework for future studies investigating metabolic regulation and nutrition‐based therapeutic strategies.

## 2 Results

### 2.1 Glycolysis‐Related Molecular Alterations Are Consistently Identified in PCOS

**FIGURE 1:**
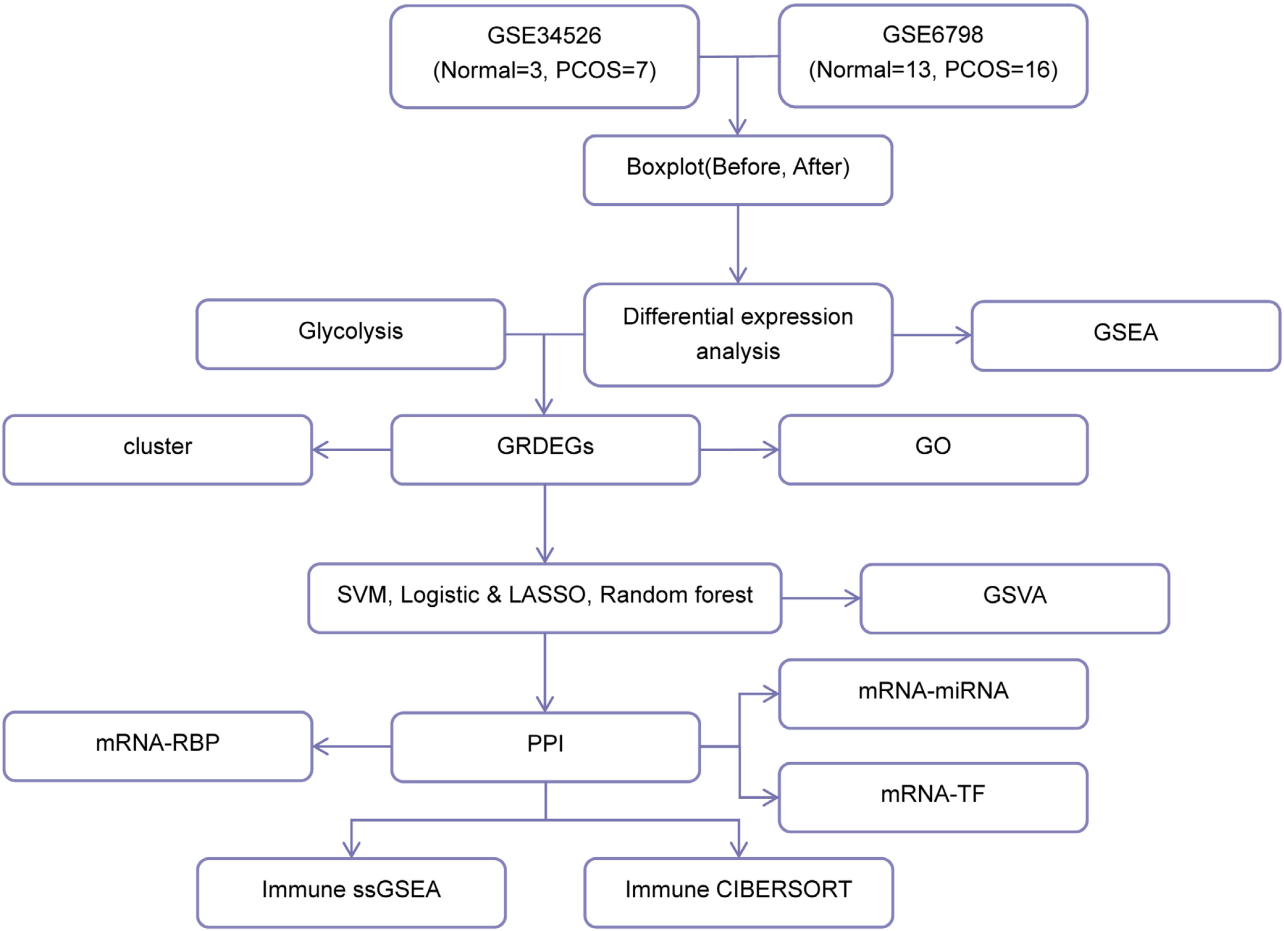
Technology roadmap. PCOS, Polycystic Ovary Syndrome; DEGs, Differentially Expressed Genes; GRDEGs, Glycolysis‐Related Differentially Expressed Genes; PPI, Protein–Protein Interaction; RBP, RNA Binding Protein; TF, Transcription Factors; GO, Gene Ontology; ssGSEA, Single‐Sample Gene‐Set Enrichment Analysis.

**FIGURE 2:**
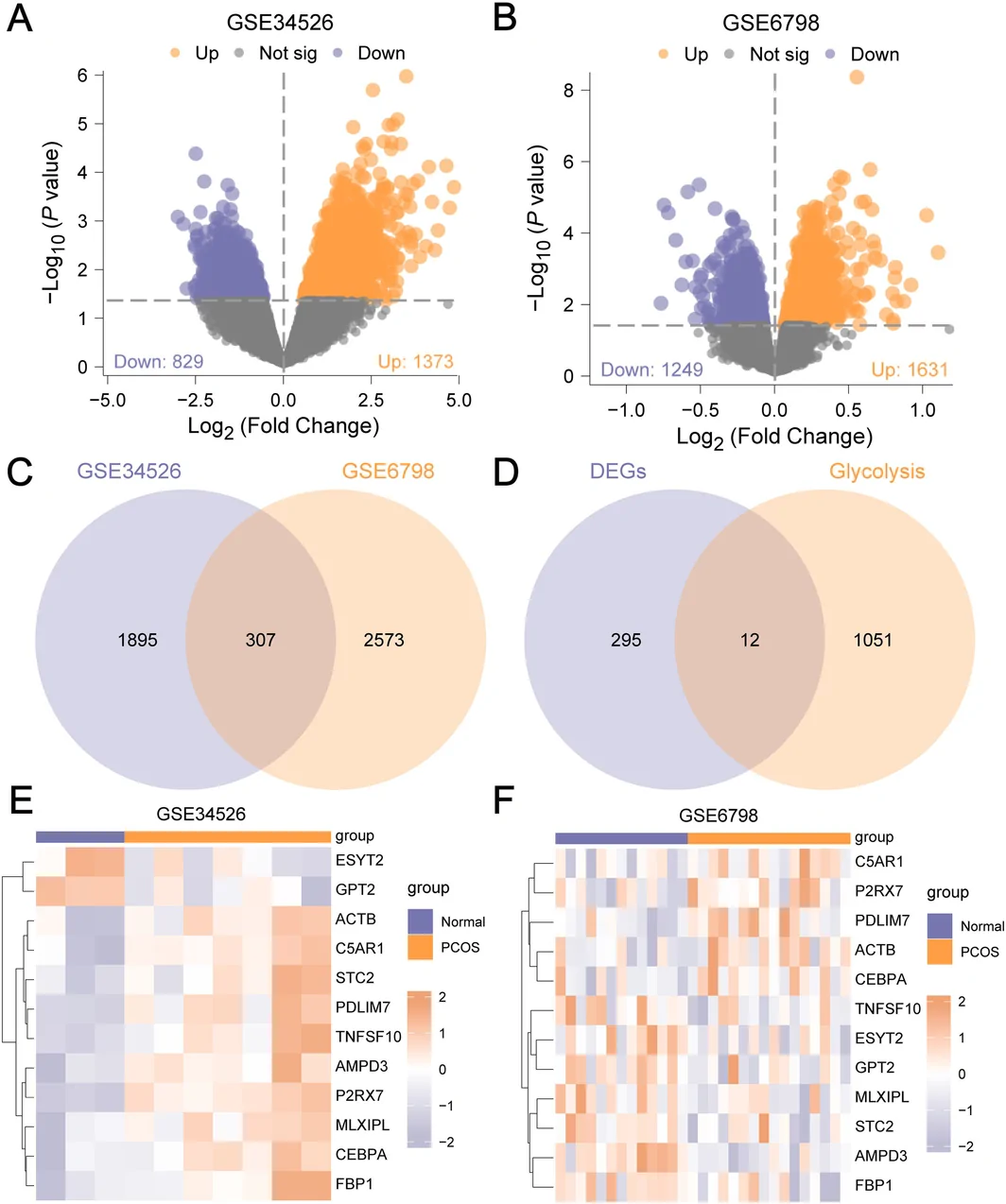
Differential expression analysis and identification of glycolysis‐related differentially expressed genes (GRDEGs) in datasets GSE34526 and GSE6798. (A) Volcano plot of differential gene analysis for Polycystic Ovary Syndrome samples (group: PCOS) and control samples (group: Normal) from dataset GSE34526. Orange, purple, and gray dots represent upregulated, downregulated, and non‐significantly changed genes, respectively. (B) Volcano plot of differential gene analysis for Polycystic Ovary Syndrome samples (group: PCOS) and control samples (group: Normal) from dataset GSE6798. Orange, purple, and gray dots represent upregulated, downregulated, and non‐significantly changed genes, respectively. (C) Venn diagram of differentially expressed genes (DEGs) in datasets GSE345526 and GSE6798. (D) Venn diagram of co‐differentially expressed genes and GRGs. (E) Heat map of glycolysis‐related differentially expressed genes (GRDEGs) in dataset GSE34526. (F) Heat map of glycolysis‐related differentially expressed genes (GRDEGs) in dataset GSE6798. PCOS: Polycystic Ovary Syndrome; GRGs: Glycolysis‐related genes; GRDEGs: Glycolysis‐related differentially expressed genes.

To identify glycolysis‐related molecular alterations that are consistently associated with PCOS across metabolically relevant tissues, two independent transcriptomic datasets derived from granulosa cells (GSE34526) and skeletal muscle (GSE6798) were incorporated into the analytical workflow (Figure 1). To minimize technical variation, batch effects between GSE34526 and GSE6798 were removed prior to downstream analyzes, resulting in comparable expression distributions across the two datasets (Figure S1). Differential expression analysis was then performed independently in each dataset to identify PCOS‐associated transcriptional changes while minimizing potential bias introduced by tissue‐specific expression patterns. Using the predefined differential expression criteria, 2202 DEGs were identified in GSE34526, including 1373 upregulated and 829 downregulated genes (Figure 2A), whereas 2880 DEGs, comprising 1631 upregulated and 1249 downregulated genes, were detected in GSE6798 (Figure 2B). To identify transcriptional alterations consistently associated with PCOS, only genes shared between the two datasets were retained for subsequent analyzes. This comparison identified 307 common DEGs (Figure 2C). Given that the primary objective of this study was to characterize glycolytic dysregulation in PCOS, the 307 shared DEGs were further intersected with a curated glycolysis‐related gene set. This analysis identified 12 glycolysis‐related differentially expressed genes (GRDEGs), namely P2RX7, C5AR1, PDLIM7, CEBPA, TNFSF10, MLXIPL, AMPD3, FBP1, GPT2, ACTB, ESYT2, and STC2 (Figure 2D).

**FIGURE 3:**
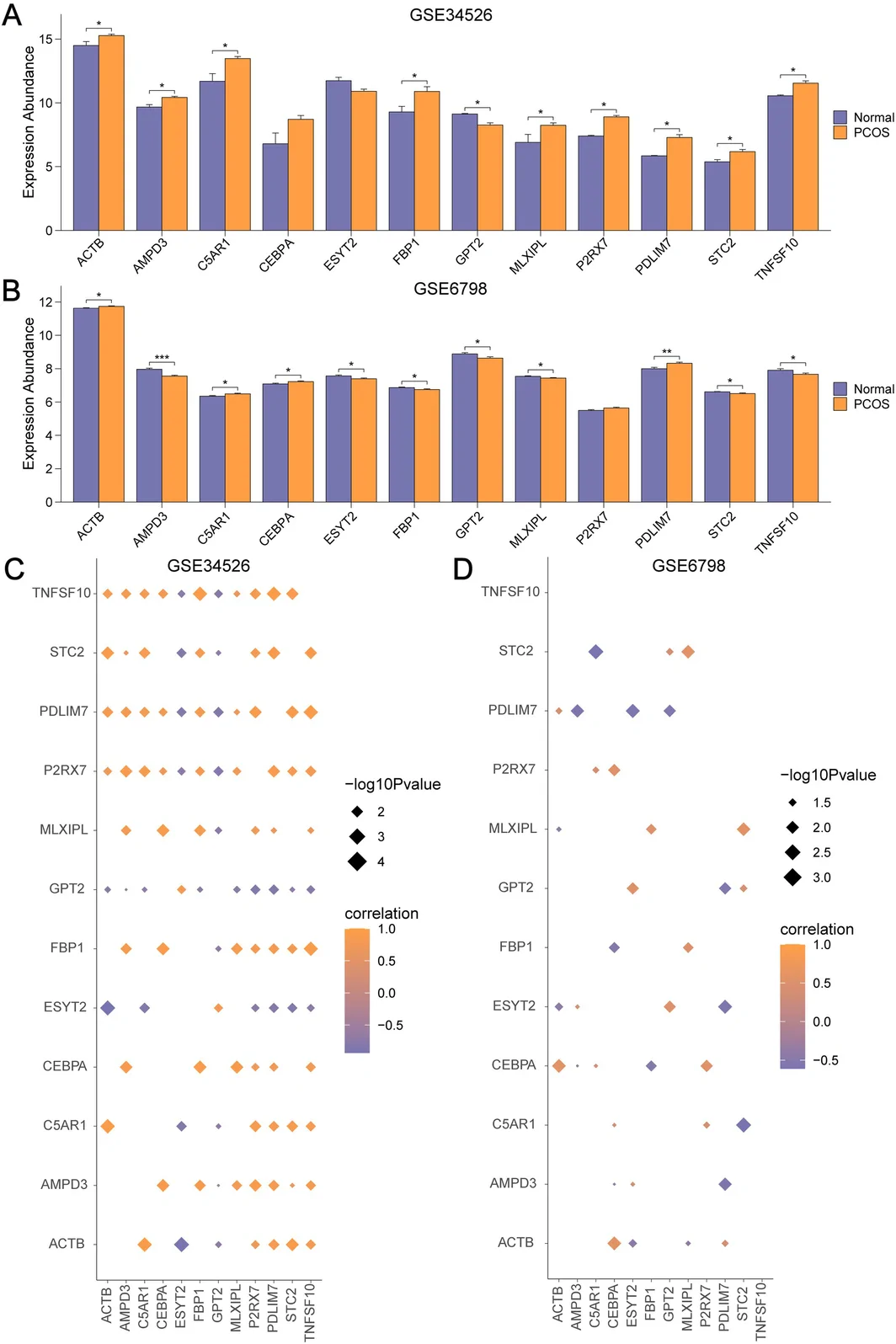
Validation and correlation analysis of glycolysis‐related differentially expressed genes (GRDEGs) in datasets GSE34526 and GSE6798. (A) Group comparison diagram of GRDEGs in the PCOS group and Normal group of the dataset GSE34526. (B) Group comparison diagram of GRDEGs in the PCOS group and Normal group of the dataset GSE6798. (C) Correlation matrix showing the pairwise expression correlations among GRDEGs in dataset GSE34526. Circle color indicates the direction of the correlation, and circle size reflects the correlation coefficient. (D) Correlation matrix showing the pairwise expression correlations among GRDEGs in dataset GSE6798. Circle color indicates the direction of the correlation, and circle size reflects the correlation coefficient. PCOS, Polycystic Ovary Syndrome; GRDEGs, glycolysis‐related differentially expressed genes. The symbol ns is equivalent to *p* value ≥ 0.05 and has no statistical significance; the symbol * is equivalent to *p* value < 0.05; the symbol ** is equivalent to *p* value < 0.01; the symbol *** is equivalent to *p* value < 0.001.

To further evaluate these GRDEGs, their expression patterns were compared between PCOS and control samples in both datasets. Heatmap analysis demonstrated distinct expression profiles of the 12 GRDEGs between the two groups (Figure 2E,F). Consistently, 10 GRDEGs were significantly differentially expressed in GSE34526, whereas 11 GRDEGs showed significant expression changes in GSE6798 (Figure 3A,B). Correlation analysis further revealed coordinated expression among these genes in both datasets. Most GRDEGs were positively correlated in GSE34526, whereas both positive and negative correlations were observed in GSE6798, suggesting a more complex correlation structure (Figure 3C,D).

Collectively, these analyzes identified a reproducible set of glycolysis‐related genes consistently dysregulated across independent PCOS datasets, providing the basis for subsequent functional characterization and prioritization of key glycolysis‐associated regulators.

### 2.2 GO Enrichment Reveals Metabolic Reprogramming Associated With Glycolytic Dysregulation

**FIGURE 4:**
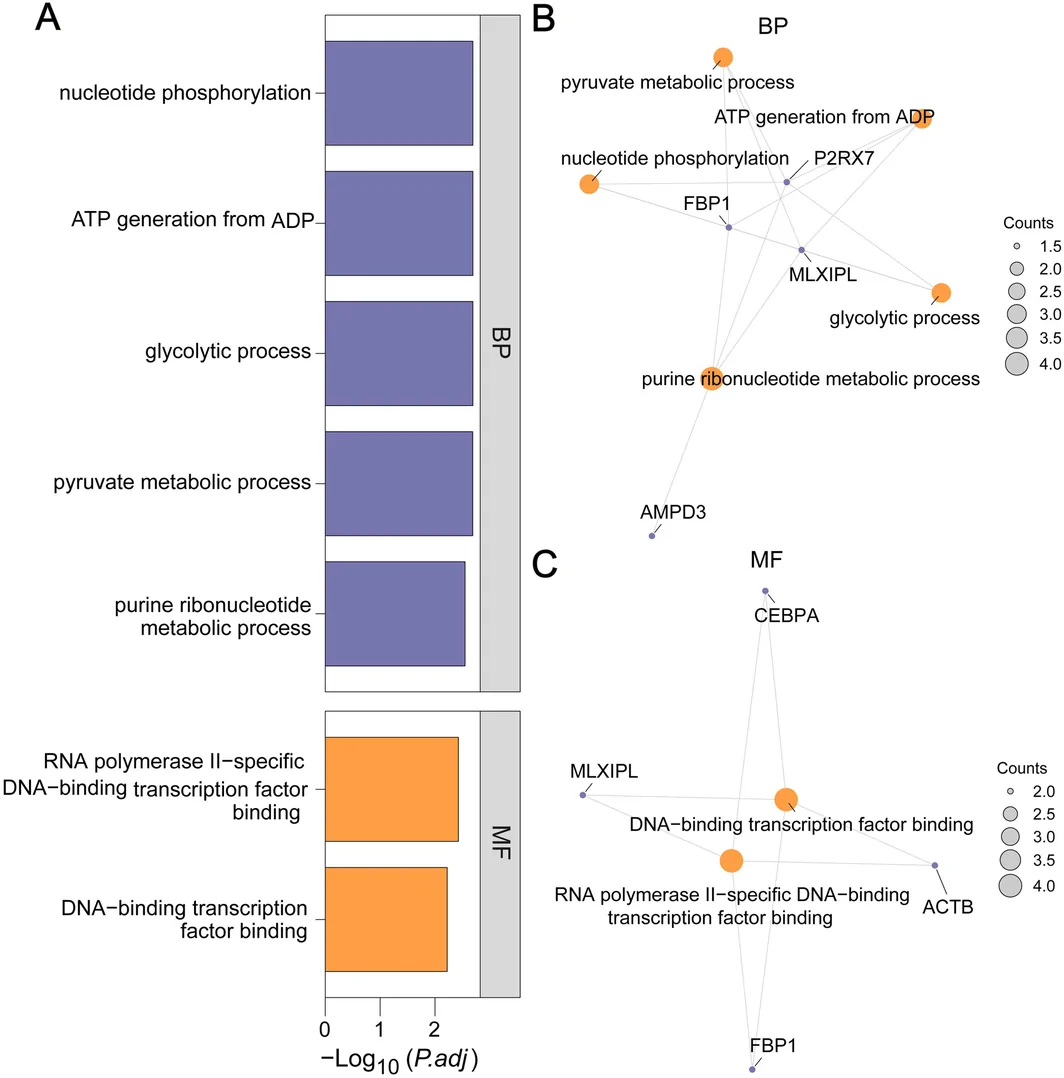
GO enrichment analysis of GRDEGs. (A) Bar plots showing the enriched biological process (BP) and molecular function (MF) terms of the 12 GRDEGs. The x‐axis represents −log10 (adjusted *p* value). (B, C) Network graph depicting the Gene Ontology (GO) results of GRDEGs: BP (B), MF (C). Orange nodes signify entries, purple nodes denote molecules, and connecting lines indicate the relationship between entries and molecules. Node size represents the number of genes associated with each GO term. PCOS, Polycystic Ovary Syndrome; GO: Gene Ontology; BP, biological process; MF, molecular function. The screening criteria for Gene Ontology (GO) enrichment analysis are *p* adj < 0.05 and FDR value (*q* value) < 0.05.

Having identified a reproducible set of glycolysis‐related differentially expressed genes (GRDEGs), we next sought to characterize the biological processes associated with these genes. Gene Ontology (GO) enrichment analysis was therefore performed to determine whether the identified GRDEGs were preferentially involved in specific biological functions relevant to PCOS. Biological process (BP) analysis showed that the GRDEGs were predominantly enriched in glycolysis‐related metabolic processes, including the glycolytic process, ATP generation from ADP, pyruvate metabolic process, nucleotide phosphorylation, and purine ribonucleotide metabolic process (Figure 4A; Table S1). These enriched terms consistently pointed to pathways involved in cellular energy production and metabolic regulation, suggesting that alterations in glycolytic metabolism represent a major transcriptional feature of PCOS. In addition to metabolic processes, molecular function (MF) analysis revealed significant enrichment in RNA polymerase II‐specific DNA‐binding transcription factor binding and DNA‐binding transcription factor binding (Figure 4A), indicating that transcriptional regulatory activities may contribute to the altered expression of glycolysis‐related genes. To further illustrate the relationships between enriched biological terms and their corresponding genes, GO enrichment networks were constructed for both BP and MF categories (Figure 4B,C). The network analysis demonstrated that multiple GRDEGs contributed simultaneously to several enriched GO terms, highlighting the interconnected nature of glycolytic metabolism and transcriptional regulation rather than independent alterations in individual genes.

Overall, GO enrichment analysis indicates that the glycolysis‐related transcriptional alterations identified in PCOS are primarily associated with metabolic reprogramming accompanied by coordinated transcriptional regulation.

### 2.3 GSEA Enrichment Analysis Further Characterizes Biological Alterations in PCOS

To further characterize biological pathways associated with PCOS, gene set enrichment analysis (GSEA) was performed in GSE34526 and GSE6798 (Figure S2; Tables S2–S4). In GSE34526, significantly enriched pathways were mainly associated with lysosome, hematopoietic cell lineage, apoptosis, and pyroptosis (Figure S2A–E). In GSE6798, enriched pathways were primarily related to mitochondrial translation, fatty acid metabolism, neutrophil degranulation, and microRNA targets in extracellular matrix and membrane receptors (Figure S2F–J).

Although distinct pathways were enriched in the two datasets, both analyzes consistently identified alterations involving metabolic and immune‐related biological processes. These results further delineate the biological pathways associated with PCOS and provide the basis for subsequent identification of key glycolysis‐related molecular signatures.

### 2.4 Machine‐Learning Identifies Candidate Glycolysis‐Associated Molecular Signatures in PCOS

**FIGURE 5:**
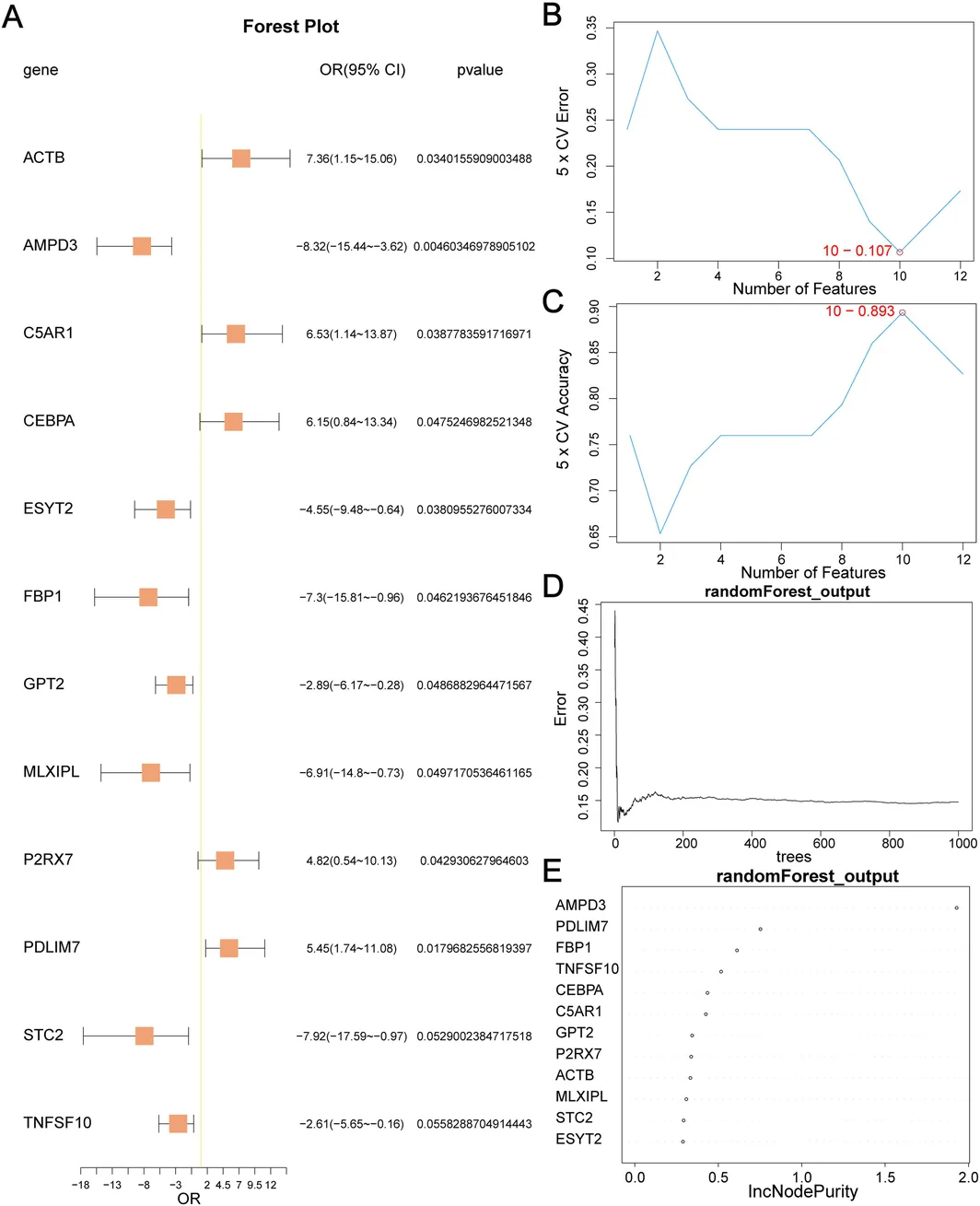
Logistic regression, SVM and random forest analysis. (A) Forest plot of logistic regression analysis of 12 GRDEGs. (B) Cross‐validation error curve of the SVM model showing the optimal number of features with the lowest error rate. (C) Cross‐validation accuracy curve of the SVM model showing the optimal number of features with the highest classification accuracy. (D) Model training error plot of the random forest algorithm. (E) Random forest model showing differential genes (in descending order of IncNodePurity). PCOS, Polycystic Ovary Syndrome; SVM, Support Vector Machine.

To identify glycolysis‐related genes with potential biological and classification relevance, machine‐learning algorithms were applied to prioritize candidate genes from the 12 GRDEGs identified in the previous analyzes. Logistic regression analysis first evaluated the association of each GRDEG with PCOS, and the expression patterns of all candidate genes are summarized in the forest plot (Figure 5A).

**FIGURE 6:**
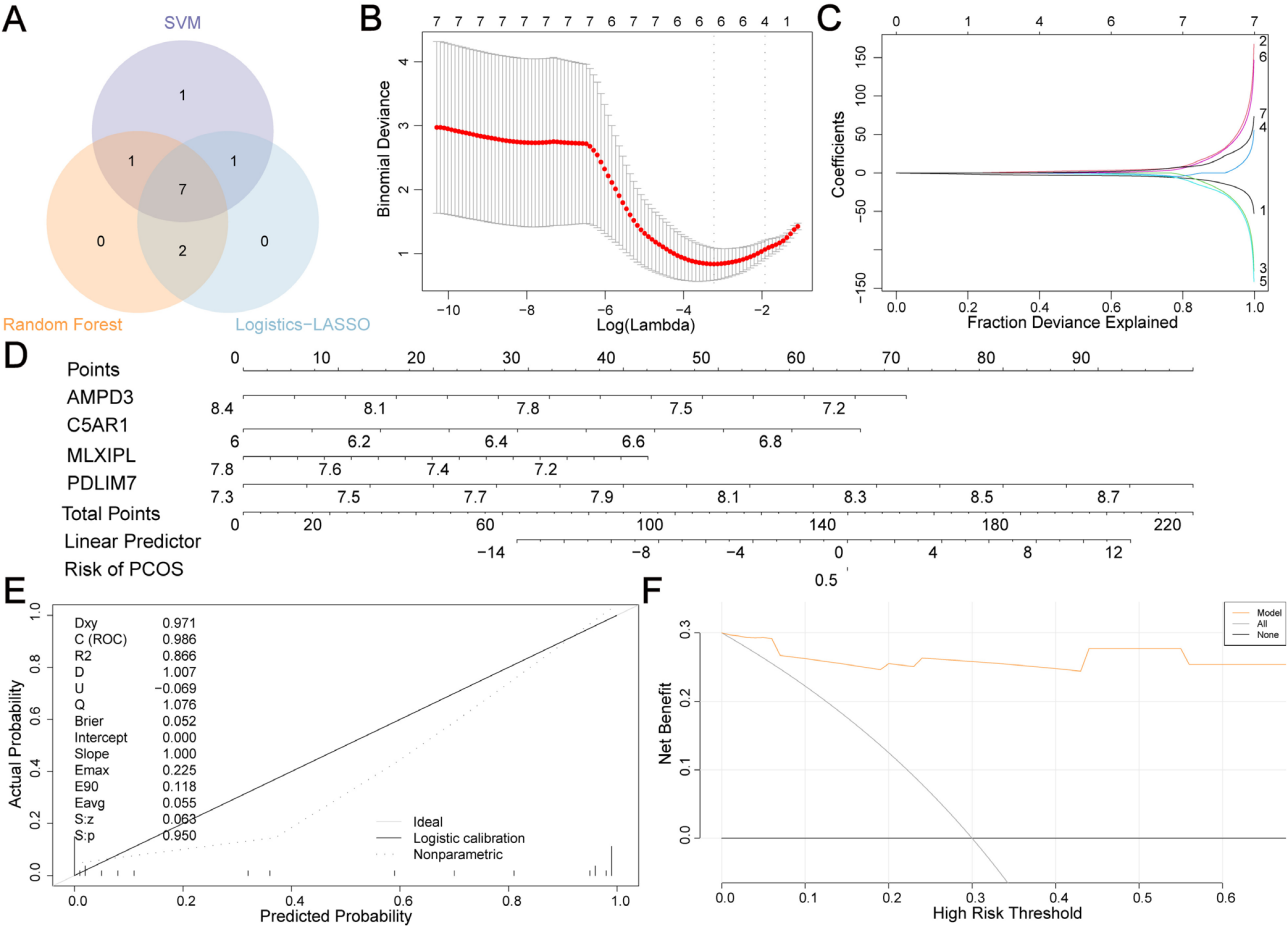
Identification and exploratory evaluation of candidate glycolysis‐related molecular signatures. (A) Venn diagram showing the intersection of candidate genes retained by logistic regression, support vector machine (SVM), and random forest analyzes. (B) LASSO regression analysis for candidate feature selection. (C) Variable trajectory plot of the LASSO regression analysis. (C) Variable trajectory diagram of the LASSO regression model. (D) Nomogram illustrating the relative contributions of the 4 hub genes to the candidate molecular signature. (E) Calibration curve showing agreement between predicted and observed sample classifications within the analyzed dataset. (F) Decision curve analysis providing an exploratory assessment of the classification performance of the candidate molecular signature across different threshold probabilities. PCOS, polycystic ovary syndrome; SVM, support vector machine; LASSO, least absolute shrinkage and selection operator; DCA, decision curve analysis.

To improve the robustness of candidate gene selection, support vector machine (SVM) and random forest (RF) analyzes were subsequently performed. The SVM model achieved the highest classification accuracy when 10 genes were included (Figure 5B,C). Similarly, RF analysis identified 10 genes with an IncNodePurity value greater than 0.2 (Figure 5D,E). Intersecting the genes retained by logistic regression, SVM, and RF yielded seven common candidate genes, including PDLIM7, AMPD3, FBP1, MLXIPL, P2RX7, CEBPA, and C5AR1 (Figure 6A).

To further refine the candidate molecular signature, LASSO regression analysis was performed using these seven candidate genes. Four genes (AMPD3, C5AR1, MLXIPL, and PDLIM7) were ultimately retained as candidate glycolysis‐related molecular signatures (Figure 6B,C). Based on the expression of these 4 hub genes, a risk score was calculated for each sample, and patients were subsequently stratified into high‐ and low‐risk groups.

To further characterize the performance of this candidate molecular signature, a nomogram was constructed using the 4 hub genes (Figure 6D). Among them, PDLIM7 contributed the greatest weight within the molecular signature. Calibration analysis demonstrated good agreement between predicted and observed classifications within the analyzed datasets (Figure 6E), while decision curve analysis suggested potential classification value of the molecular signature within the analyzed datasets across a broad range of threshold probabilities (Figure 6F).

**FIGURE 7:**
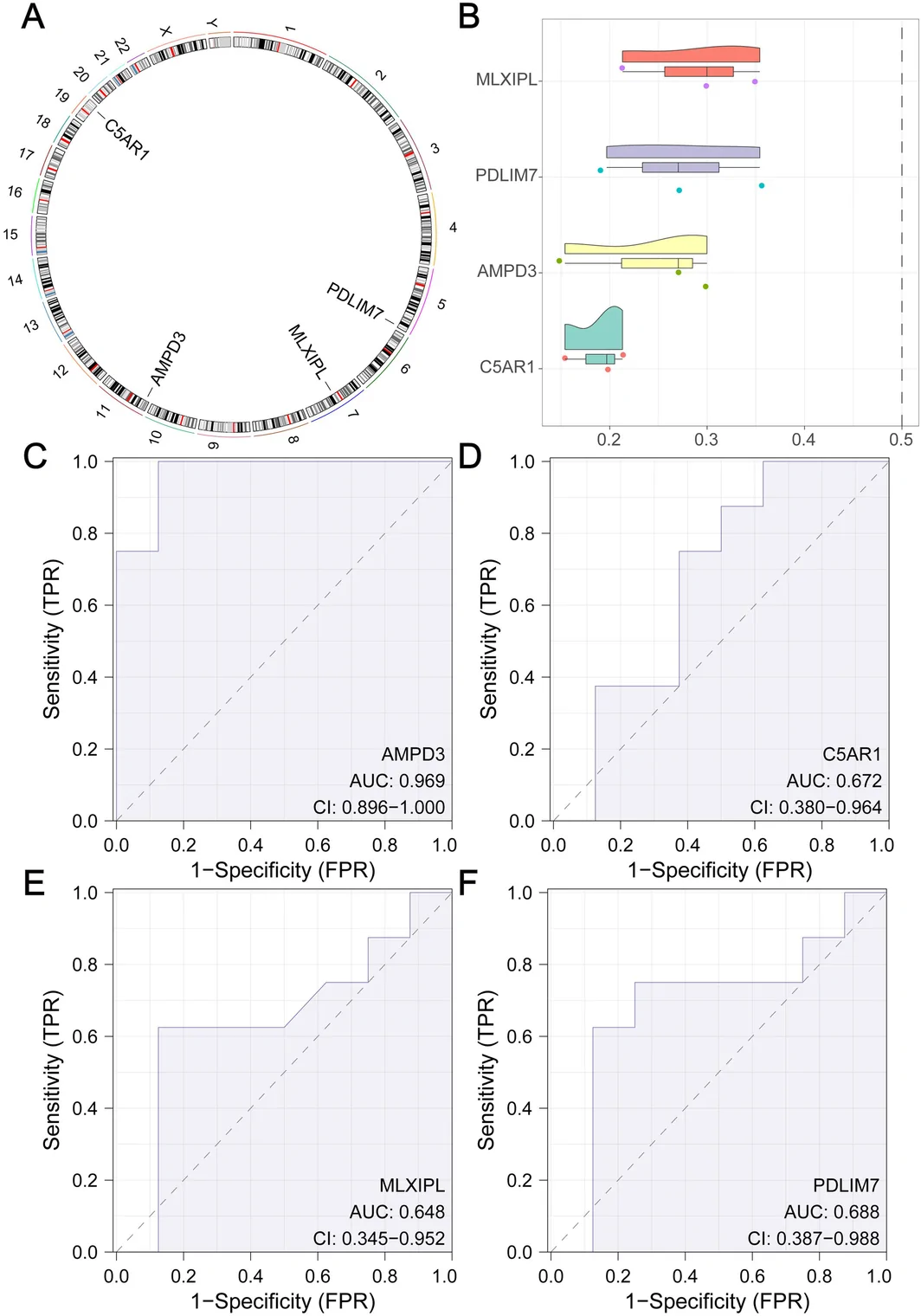
Characterization and exploratory classification performance of hub GRDEGs. Chromosomal locations of the 4 hub genes (A). Functional similarity analysis of the hub genes (B). Receiver operating characteristic (ROC) curves for AMPD3 (C), C5AR1 (D), MLXIPL (E), and PDLIM7 (F) in the GSE6798 dataset (C–F). ROC curves and corresponding areas under the curve (AUCs) were used to characterize the discriminatory performance of individual hub genes in distinguishing PCOS from control samples within the analyzed dataset. PCOS, polycystic ovary syndrome; ROC, receiver operating characteristic; AUC, area under the curve.

The 4 hub genes were further characterized by their chromosomal distribution and functional similarity. Chromosomal mapping showed that AMPD3, C5AR1, MLXIPL, and PDLIM7 were located on chromosomes 5, 7, 11, and 19, respectively (Figure 7A). Functional similarity analysis identified MLXIPL as the gene with the highest functional similarity score among the 4 hub genes (Figure 7B).

Finally, the discriminatory ability of each hub gene was evaluated by receiver operating characteristic (ROC) analysis. Among the four genes, AMPD3 exhibited the highest discriminatory ability (AUC = 0.90), whereas C5AR1, MLXIPL, and PDLIM7 showed comparatively lower discriminatory performance (Figure 7C–F).

### 2.5 GSVA Reveals Distinct Pathway Activities Associated With Glycolysis‐Related Molecular Signatures

**FIGURE 8:**
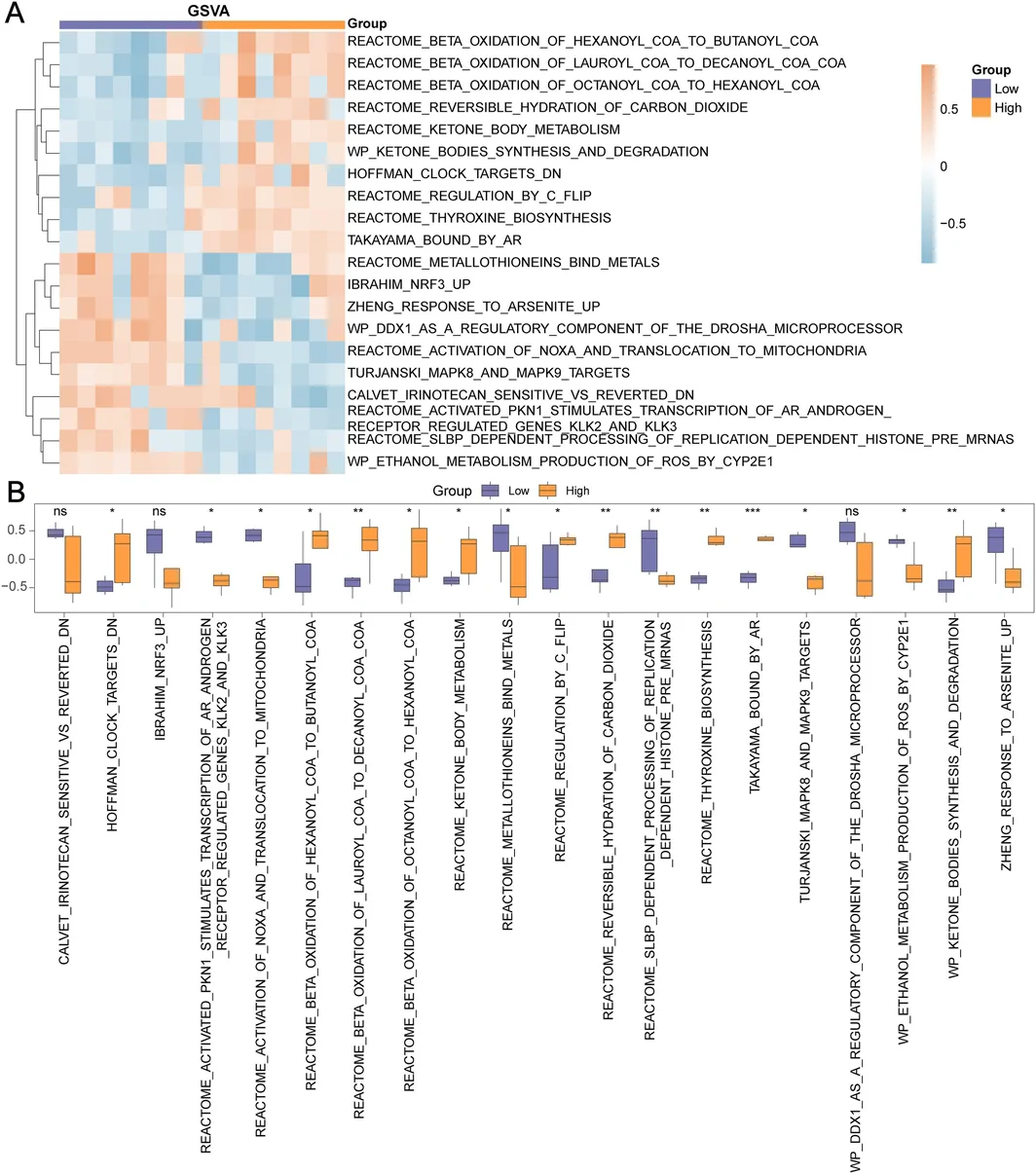
GSVA enrichment analysis according to the candidate molecular‐signature score in GSE6798 (A, B). GSVA results comparing the high‐ and low‐score groups defined by the median classification score of the candidate molecular signature, presented as a heatmap (A) and box plots (B). The heatmap shows the enrichment scores of significantly altered pathways, and the box plots compare pathway enrichment scores between the two groups. ns, *p* value ≥ 0.05; *p value < 0.05; **p value < 0.01; ***p value < 0.001. The screening criterion for gene set variation analysis (GSVA) was *p* value < 0.05.

To characterize the biological features associated with glycolysis‐related molecular signatures in PCOS, gene set variation analysis (GSVA) was performed using the 4 hub genes identified by machine‐learning analysis. Samples were stratified into high‐ and low‐risk groups according to the calculated risk score, and pathway activities were subsequently compared between the two groups (Table S2; Figure 8).

The GSVA heatmap demonstrated distinct pathway activity patterns between the high‐ and low‐risk groups (Figure 8A). Differential pathway analysis further identified widespread alterations involving metabolic, inflammatory, and endocrine‐related pathways (Figure 8B). Specifically, pathways associated with fatty acid β‐oxidation, ketone body metabolism, pyruvate metabolism, and mitochondrial function were significantly altered, indicating extensive metabolic remodeling. In addition, multiple immune‐ and stress‐related pathways, including interferon signaling, IL6 signaling, reactive oxygen species signaling, hypoxia, and TGF‐β signaling, also differed between the two groups. Alterations were likewise observed in pathways associated with androgen response, estrogen response, and xenobiotic metabolism, suggesting that glycolysis‐related molecular signatures are accompanied by broad biological changes extending beyond energy metabolism.

Together, these results demonstrate that the glycolysis‐related molecular signatures identified in PCOS are associated with coordinated alterations in metabolic, immune, and endocrine‐related biological pathways.

### 2.6 Regulatory Network Analysis Suggests Multilayered Regulation of Glycolysis‐Related Hub Genes

**FIGURE 9:**
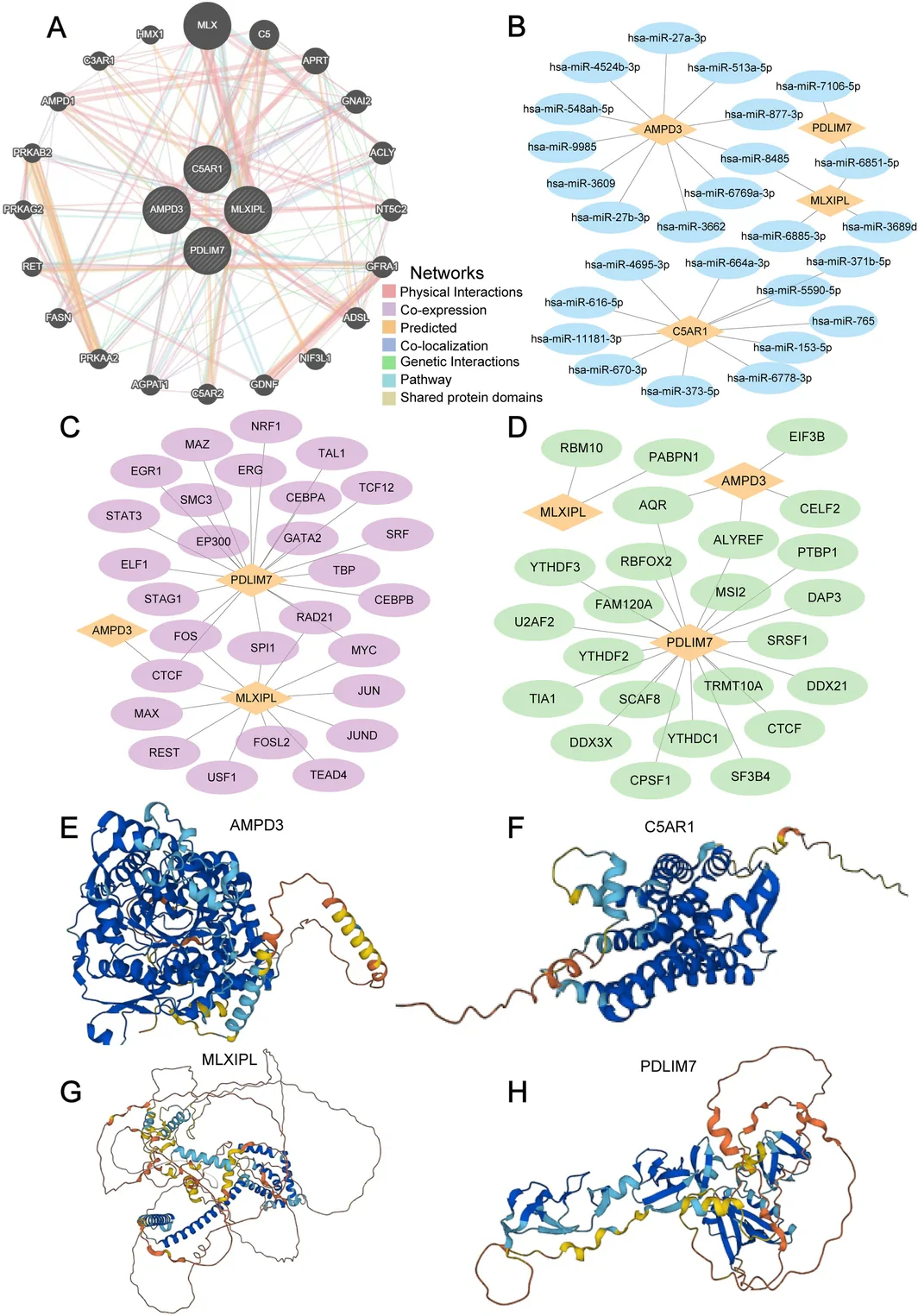
Construction of mRNA‐miRNA, mRNA‐TF, and mRNA‐RBP interaction network. GeneMANIA functional association network of the 4 hub genes. (B) The mRNA—miRNA regulatory network of the hub gene. (C) The mRNA—TF regulatory network of the hub gene. (D) The mRNA—RBP regulatory network of the hub gene. Protein structure display of the hub gene (AMPD3 (E), C5AR1 (F), MLXIPL (G), PDLIM7 (H)). PPI network, protein—protein interaction network; TF, transcription factors; RBP, RNA binding protein. Orange represents mRNA, purple represents TF, blue represents miRNA, and green represents RBP.

The GeneMANIA database (http://genemania.org) serves as a valuable resource for analyzing, predicting, and identifying gene functions within an input gene list to identify and prioritize genes. We utilized the GeneMANIA database to investigate the correlation between hub genes and other genes (Figure 9A). The results demonstrate that hub genes primarily interact with other genes through physical interactions (Physical Interactions), co‐expression (Co‐expression), prediction (Predicted), co‐localization (Co‐localization), genetic interactions (Genetic Interactions), pathway involvement, and shared protein domains.

Subsequently, miRNAs associated with hub genes were identified using the miRDB database. The mRNA‐miRNA regulatory network was constructed and visualized via Cytoscape software (Figure 9B). This network contains 4 hub genes and 26 miRNAs. Detailed information is available in Table S5.

Next, transcription factors (TF) interacting with hub genes were identified via the ChIPBase database. The mRNA‐TF regulatory network was constructed and visualized using Cytoscape software (Figure 9C), comprising 3 hub genes and 28 transcription factors. Detailed information is available in Table S6.

Thereafter, RNA‐binding proteins (RBPs) associated with the hub genes were predicted using the StarBase database. The mRNA‐RBP regulatory network was then constructed and visualized in Cytoscape software (Figure 9D). This network comprises 3 hub genes and 24 RNA‐binding proteins (RBPs). Detailed information is available in Table S7.

Finally, the AlphaFold Protein Structure Database (https://www.alphafold.ebi.ac.uk/) contains nearly 350,000 protein structures predicted by the AlphaFold artificial intelligence system. These structures cover humans and 20 commonly used model organisms in biological research ( *E. coli* , Drosophila, zebrafish, mice, etc.). For the human proteome specifically, AlphaFold predicted the structure of 98.5% of human proteins. We utilized the AlphaFold website to analyze the protein structures of the hub genes and display the results (Figure 9E–H).

### 2.7 Glycolysis‐Related Molecular Signatures Are Associated With Immune Remodeling

**FIGURE 10:**
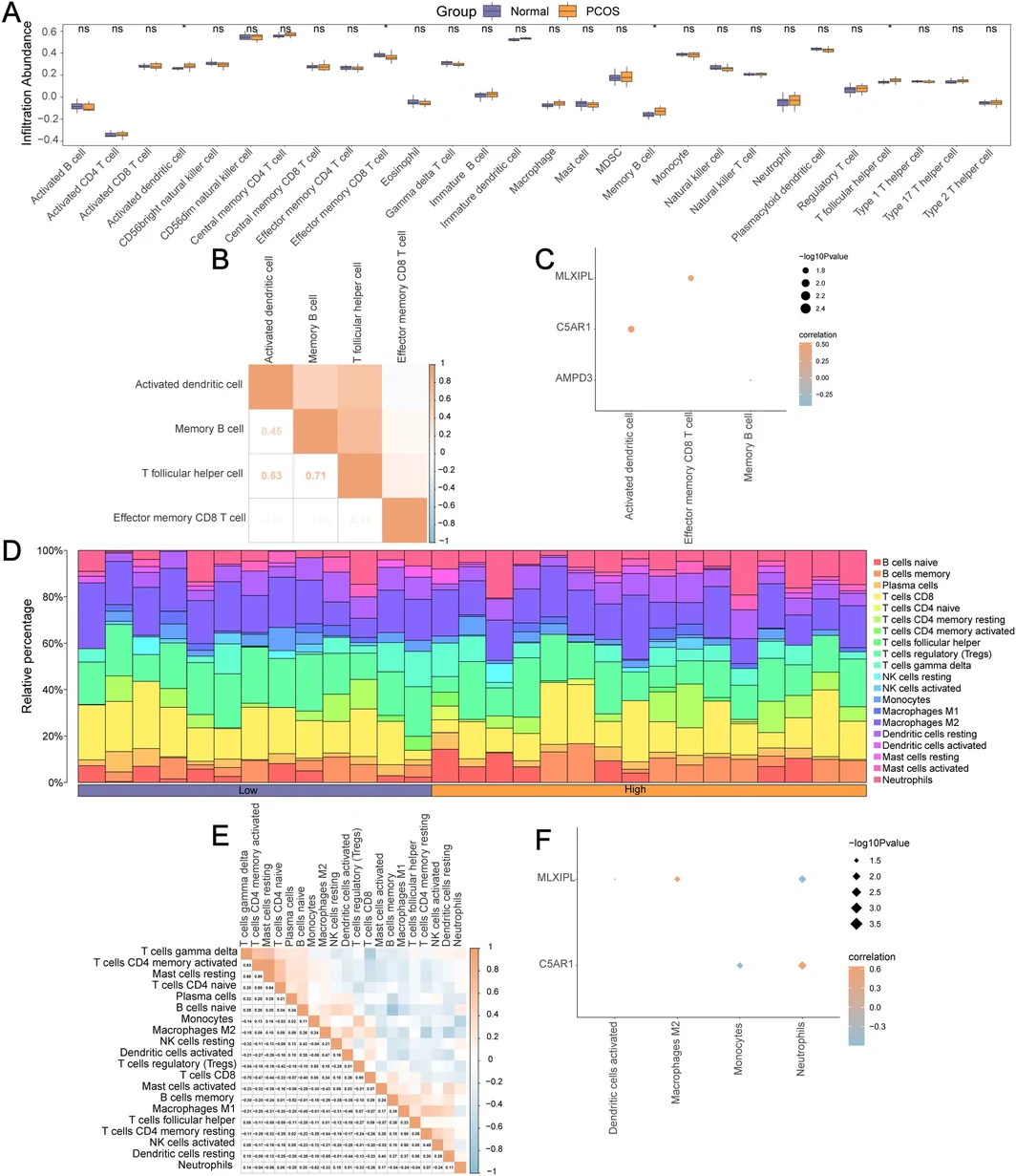
Immune infiltration analysis of data set GSE6798. (A) Comparison of ssGSEA immune cell infiltration scores between the PCOS and control groups in data set GSE6798. (B) Correlation analysis results of immune cell infiltration abundance in the data set GSE6798. The color scale indicates the correlation coefficient. (C) Bubble diagram of the correlation between immune cells and hub genes in the data set GSE6798. Circle color indicates the direction of the correlation, circle size reflects the correlation strength, and asterisks indicate statistical significance. (D) Histogram of the CIBERSORT immune infiltration analysis results of the data set GSE6798. (E) Correlation analysis results of immune cell infiltration abundance in data set GSE6798. The color scale indicates the correlation coefficient. (F) Bubble diagram of the correlation between immune cells and hub genes in the data set GSE6798. Circle color indicates the direction of the correlation, circle size reflects the correlation strength, and asterisks indicate statistical significance. The symbol ns is equivalent to *p* value ≥ 0.05 and has no statistical significance; the symbol * is equivalent to *p* value < 0.05; the symbol ** is equivalent to *p* value < 0.01; the symbol *** is equivalent to *p* value < 0.001. The value in the lower left corner of the correlation point map is the correlation coefficient (*R* value); the number in the lower left corner of the correlation heat map represents the correlation coefficient (*R* value). The absolute value of the correlation coefficient (*R*) is weak or irrelevant when it is below 0.3. The correlation between 0.3 and 0.5 is weak, the correlation between 0.5 and 0.8 is moderate, and the correlation above 0.8 is strong. PCOS, Polycystic Ovary Syndrome; ssGSEA, Single‐Sample Gene‐Set Enrichment Analysis.

Applying the ssGSEA algorithm to the expression matrix of the primary dataset GSE6798, we calculated the infiltration abundance of 28 immune cell types. First, immune cells demonstrating significant differential abundance across groups (*p* < 0.05) were identified and visualized in a group comparison chart. This analysis, presented in Figure 10A, revealed four statistically significant immune cell types (*p* < 0.05): Activated dendritic cell, Effector memory CD8 T cell, Memory B cell, and T follicular helper cell. Next, the correlation results for the infiltration abundance of these four significant immune cell types were displayed in a correlation heatmap (Figure 10B), revealing strong positive correlations among most cells. Finally, a correlation bubble chart (Figure 10C) illustrated the relationships between hub genes and immune cell infiltration abundance. These results demonstrate a significant positive correlation between the hub genes C5AR1 and MLXIPL and the immune cells (Activated dendritic cell and Effector memory CD8 T cell, respectively). Conversely, a significant negative correlation exists between the hub gene AMPD3 and Memory B cells.

We leveraged the expression matrix from the primary dataset GSE6798 to compute correlations for 22 distinct immune cell types between the PCOS group and the Normal group, utilizing the CIBERSORT algorithm. Building upon the immune infiltration analysis results, we generated a histogram depicting the proportional distribution of immune cells within the GSE6798 dataset (Figure 10D). These 20 immune cell types encompass: B cells naïve, B cells memory, Plasma cells, T cells CD8, T cells CD4 naïve, T cells CD4 memory resting, T cells CD4 memory activated, T cells follicular helper, T cells regulatory (Tregs), T cells gamma delta, NK cells resting, NK cells activated, Monocytes, Macrophages M1, Macrophages M2, Dendritic cells resting, Dendritic cells activated, Mast cells resting, Mast cells activated, and Neutrophils. Subsequently, we visualized the correlation outcomes between the infiltration abundance of these 20 immune cell types through a correlation point plot (Figure 10E). This revealed that the majority exhibited negative correlations. Finally, we illustrated the relationship between hub genes and immune cell infiltration abundance in the GSE6798 dataset using a correlation bubble chart (Figure 10F). Strikingly, the bubble chart results demonstrate a significant positive correlation between the hub gene MLXIPL and immune cells (Dendritic cells activated, Macrophages M2, Neutrophils); conversely, a significant negative correlation exists between the hub gene C5AR1 and immune cells (Monocytes, Neutrophils).

### 2.8 GPT2 Regulates Glycolytic Metabolism and Metabolic Homeostasis in Granulosa Cells

**FIGURE 11:**
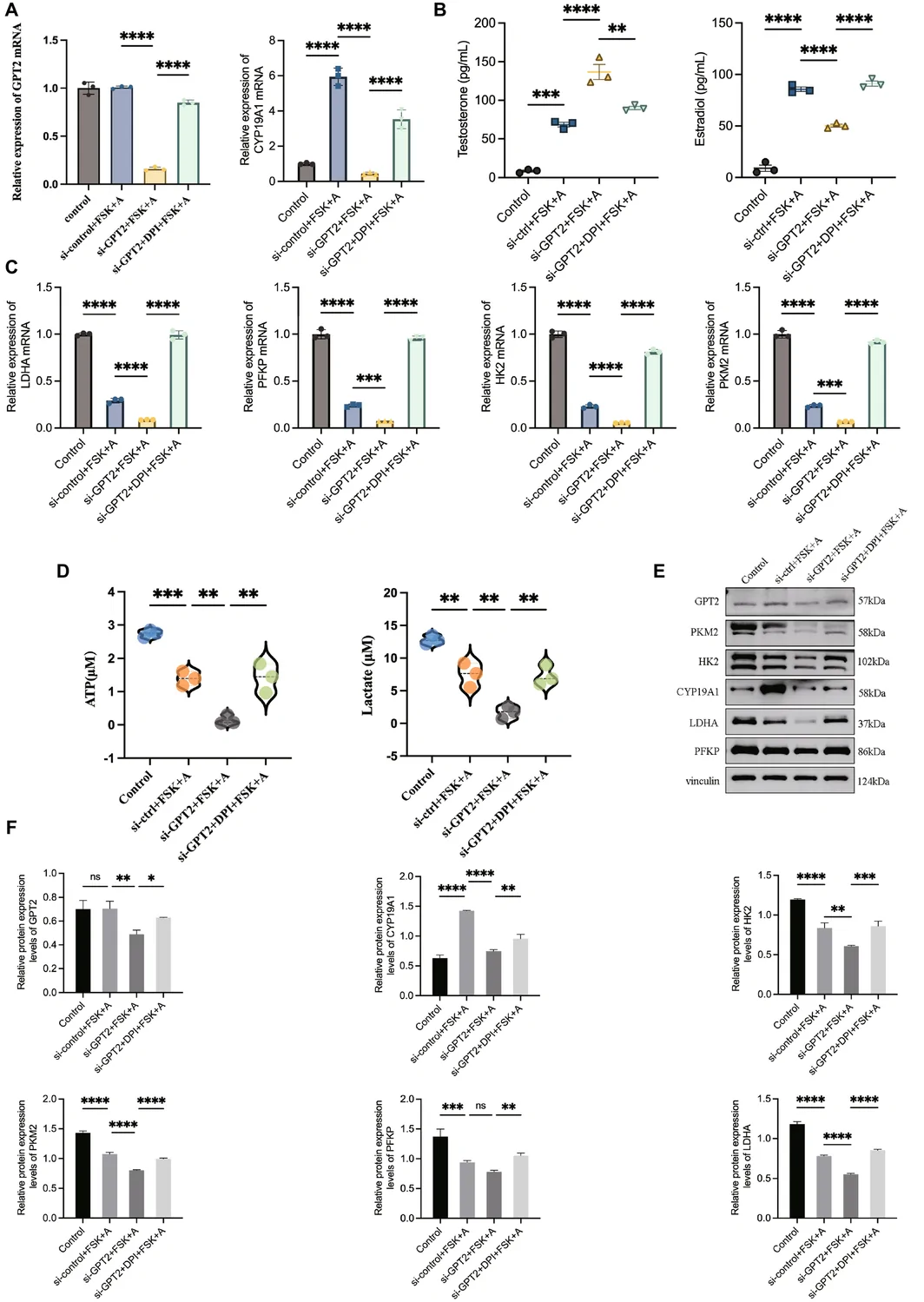
GPT2 involvement in glycolysis in PCOS. (A) Relative mRNA expression levels of GPT2 and CYP19A1 in KGN granulosa cells following GPT2 knockdown and DPI treatment. (B) Testosterone and estradiol concentrations in the culture supernatant under different treatment conditions. (C) Relative mRNA expression levels of glycolysis‐related genes (LDHA, PFKP, HK2, and PKM2) in KGN granulosa cells. (D) Intracellular ATP and lactate levels following GPT2 knockdown and DPI treatment. (E) Representative western blot images showing the protein expression of GPT2, CYP19A1, HK2, PKM2, LDHA, and PFKP, with vinculin as the loading control. (F) Quantitative analysis of the protein expression levels shown in panel E. Control: Cells cultured in phenol red‐free DMEM/F12 supplemented with 10% fetal bovine serum (FBS) without transfection or drug treatment. si‐control + FSK + A: Cells transfected with negative control siRNA and treated with forskolin (FSK) and testosterone (A). si‐GPT2 + FSK + A: Cells transfected with GPT2 siRNA and treated with FSK and testosterone. si‐GPT2 + DPI + FSK + A: Cells transfected with GPT2 siRNA and treated with DPI, FSK, and testosterone. Data are presented as mean ± SD from three independent biological replicates. One‐way analysis of variance (ANOVA) followed by Tukey's multiple‐comparisons test was used for statistical analysis. ns, *p* ≥ 0.05; *p < 0.05; **p < 0.01; ***p < 0.001; ****p < 0.0001.

While the machine learning analyzes identified AMPD3, C5AR1, MLXIPL, and PDLIM7 as candidate molecular signatures with diagnostic potential for PCOS, these analyzes were intended to prioritize biomarkers for disease classification rather than mechanistic investigation. GPT2 was selected independently for functional validation because it was consistently differentially expressed across datasets and has a well‐established role in glycolytic metabolism, making it a biologically relevant candidate for investigating metabolic dysfunction in PCOS. To investigate the functional role of GPT2 in granulosa‐cell metabolism, GPT2 was silenced in KGN cells, followed by treatment with the glycolysis activator diphenyleneiodonium (DPI) under PCOS‐like conditions. GPT2 knockdown efficiency was first confirmed by qRT‐PCR, which showed a marked reduction in GPT2 mRNA expression compared with the si‐control group, whereas DPI treatment partially restored GPT2 expression (Figure 11A).

We next examined whether GPT2 deficiency affected steroidogenic function. Silencing GPT2 significantly decreased CYP19A1 mRNA expression, accompanied by increased testosterone and reduced estradiol secretion, indicating impaired steroid hormone synthesis. These alterations were partially reversed following DPI treatment (Figure 11A,B).

To determine whether the impaired steroidogenic phenotype was accompanied by altered glycolytic metabolism, the expression of key glycolytic enzymes was subsequently evaluated. GPT2 knockdown markedly reduced the mRNA expression of LDHA, PFKP, HK2, and PKM2, all of which were partially restored by DPI treatment (Figure 11C). Consistently, GPT2 silencing significantly decreased both intracellular ATP and lactate levels, whereas DPI treatment partially restored ATP production and lactate content (Figure 11D).

Western blot analysis further confirmed these findings at the protein level (Figure 11E,F). Compared with the si‐control group, GPT2 knockdown reduced the protein expression of CYP19A1, HK2, PKM2, PFKP, and LDHA, whereas DPI treatment partially restored their expression. Collectively, these findings indicate that GPT2 is required for maintaining glycolytic metabolism and metabolic homeostasis in granulosa cells and that pharmacological activation of glycolysis partially alleviates the metabolic and steroidogenic abnormalities induced by GPT2 deficiency.

## 3 Discussion

Polycystic ovary syndrome is increasingly recognized as a complex endocrine‐metabolic disorder in which profound alterations in energy metabolism contribute to ovarian dysfunction and disease heterogeneity. In the present study, we systematically characterized the transcriptomic landscape of glycolysis‐associated molecular alterations in PCOS and demonstrated that dysregulated glycolytic pathways are closely linked to immune‐metabolic remodeling. By integrating transcriptomic profiling with machine learning analyzes and functional validation, we identified key glycolysis‐associated regulators and further demonstrated that impaired GPT2‐mediated glycolytic metabolism disrupts granulosa cell energy homeostasis and steroidogenic function. Collectively, these findings support glycolytic dysregulation as a fundamental feature of PCOS pathophysiology and provide new insights into the metabolic mechanisms underlying ovarian dysfunction, offering a molecular rationale for future metabolic and nutrition‐oriented therapeutic strategies.

Our study identified twelve glycolysis‐related differentially expressed genes (GRDEGs) that exhibit significant alterations in PCOS patients, underscoring their potential roles in disease pathogenesis. Among these, P2RX7, a purinergic receptor known to mediate inflammatory responses, has been implicated in various metabolic disorders, suggesting its contribution to the inflammatory component of PCOS (Di Virgilio et al. 2017). Similarly, C5AR1, a receptor for complement component C5a, plays a pivotal role in immune response and inflammation, consistent with the immune dysregulation frequently observed in PCOS patients (Butler et al. 2022). CEBPA, critical for adipocyte differentiation and lipid metabolism, links to metabolic disturbances characteristic of PCOS (Dumesic et al. 2021). TNFSF10 (also known as TRAIL) regulates apoptosis and immune function, highlighting its potential involvement in apoptotic pathway disruption in PCOS (Wang et al. 2023). MLXIPL, a transcription factor governing glycolytic gene expression, reflects the metabolic shift favoring glycolysis seen in PCOS (Shah et al. 2023). Notably, FBP1 was significantly upregulated in cumulus cells from obese PCOS patients without clinical insulin resistance, and its aberrant expression correlates directly with local glucose metabolic dysfunction in reproductive cells (Chehin et al. 2020). These findings align with existing literature emphasizing the crucial role of glycolytic metabolism in PCOS pathophysiology. Yan et al. demonstrated that downregulation of lncRNA SNHG12 in granulosa cells from PCOS patients impairs glycolysis, leading to follicular dysplasia (Yan et al. 2025) Similarly, Han et al. found that the deadenylase CNOT6L is upregulated in PCOS, inhibiting glycolysis and impairing energy supply to oocytes (Han et al. 2025).

Recent advances in PCOS research have underscored glycolysis‐related pathogenic mechanisms, validated novel biomarkers, and highlighted bioinformatics/machine learning applications in biomarker discovery. Glycolysis‐associated biomarkers TXNIP and TGFBI via public dataset analyzes, with clinical RT‐PCR validation confirming their upregulation in PCOS granulosa cells and identifying potential therapeutic compounds (Zhu et al. 2025). Gene enrichment analysis revealed that the 12 GRDEGs were significantly enriched in glycolytic process, ATP generation, and purine ribonucleotide metabolic process, along with RNA polymerase II transcription factor binding functions. GSEA results indicated abnormal enrichment in lysosome, hematopoietic cell lineage, apoptosis, mitochondrial translation, and fatty acid metabolism pathways in PCOS samples. GSVA analysis revealed imbalances in ketone body metabolism, ethanol metabolism, and beta‐oxidation. These findings support the concept that glycolytic and derived energy and nucleic acid metabolic abnormalities represent important pathological foundations of PCOS (Liang et al. 2021). Glycolysis‐related genes not only regulate local ovarian function but also participate in systemic energy metabolism, oxidative stress, and cell death pathways (Cao et al. 2025; Liu et al. 2024). This comprehensive metabolic dysregulation provides new directions for mechanistic research and targeted therapy development.

GPT2 is a mitochondrial enzyme essential for the glutamate–alanine cycle and closely linked to cellular metabolic activity (Baytas et al. 2022). Our transcriptomic and functional analyzes show that GPT2 is consistently downregulated in PCOS granulosa cells and that its deficiency impairs glycolysis, ATP production, and steroidogenesis. Beyond GPT2, our machine‐learning pipeline identified four glycolysis‐related hub genes—AMPD3, C5AR1, MLXIPL, and PDLIM7—that may functionally intersect with GPT2 within a coordinated metabolic network. MLXIPL, as a glucose‐responsive transcription factor, is positioned to link carbohydrate availability to the transcriptional control of glycolytic enzymes (Iizuka and Horikawa 2008); given that GPT2 mediates amino‐derived anaplerotic flux into the TCA cycle, we propose that MLXIPL and GPT2 jointly coordinate carbon partitioning from both glucose and amino acid sources to support granulosa cell energy demands (Baytas et al. 2022; Havula and Hietakangas 2018). C5AR1, beyond its well‐known role in inflammation, has been shown to promote glycolytic metabolism in immune cells (Li, Yang, et al. 2026), suggesting that its upregulation in PCOS ovaries may connect local immune activation to glycolytic reprogramming, potentially acting in parallel with GPT2 to modulate metabolic‐immune crosstalk. AMPD3, which catalyzes AMP deamination to regulate cellular energy charge, may couple glycolytic ATP production with nucleotide homeostasis, a function that could become particularly critical under GPT2‐deficient conditions where ATP generation is compromised (Davis et al. 2020; Miller et al. 2021). PDLIM7, a scaffolding protein with emerging roles in signal transduction, may integrate extracellular metabolic signals and influence glycolytic enzyme stability or localization, potentially cooperating with GPT2 at the post‐translational level (Li, Li, et al. 2026; Jiang et al. 2023). Collectively, these observations suggest that GPT2 and the 4 hub genes may represent complementary components of the glycolytic regulatory landscape in PCOS. While the present study did not directly investigate their functional interactions, these potential relationships warrant further mechanistic investigation in future studies.

Construction of PPI and mRNA‐miRNA, mRNA‐TF, and mRNA‐RBP interaction networks revealed prominent biological network positions for hub genes, with AlphaFold predicting protein structures. ssGSEA and CIBERSORT analyzes revealed significant abnormal infiltration of four immune cell types (Activated dendritic cells, Effector memory CD8 T cells, Memory B cells, and T follicular helper cells) in PCOS, with strong correlations between hub genes (C5AR1, MLXIPL, AMPD3) and immune cell infiltration. These findings demonstrate deep coupling between metabolic and immune microenvironments in PCOS, with hub genes influencing disease development and immune response through multi‐omics network regulation, protein interaction, and non‐coding RNA regulatory mechanisms. Previous studies have highlighted the significant role of immune dysregulation in PCOS (Banerjee et al. 2023; Qu et al. 2022). Chronic low‐grade inflammation in PCOS does not occur in isolation but forms a mutually reinforcing cycle with metabolic abnormalities through specific pathways. Among these, the IL‐17 signaling pathway and the NLRP3 inflammasome pathway serve as two key molecular mediators (Li et al. 2024; Samadi Nasab et al. 2025). Hyperandrogenism, the central endocrine characteristic of PCOS, not only affects reproductive function but also directly modulates immune cell activity, acting as an upstream trigger that disrupts immune‐metabolic homeostasis (Torstensson et al. 2024).

Although GPT2 role in the reproductive system is not well understood, transcriptomic analyzes show that it is consistently downregulated in ovarian tissues of PCOS patients and strongly correlated with key glycolytic enzymes. Using a PCOS granulosa‐cell model, we found that reduced GPT2 impairs glycolysis, energy production, and hormone synthesis, while activating glycolysis reverses these effects. These results indicate that GPT2 may be a critical metabolic regulator in PCOS and a potential link between glutamate metabolism, glycolysis, and hormonal function. From a nutritional metabolism perspective, the glutamate–alanine cycle positions GPT2 at the intersection of amino acid and carbohydrate metabolism, where it may respond to dietary carbohydrate intake and insulin resistance—both of which are known to modulate glycolytic flux in PCOS patients (Manta et al. 2023; Johnson et al. 2025). Emerging evidence has established that low‐glycemic‐index and high‐fiber diets significantly improve insulin sensitivity and metabolic profiles in PCOS (Zhang et al. 2025), while dietary patterns with high glycemic load are positively associated with PCOS risk and severity (Johnson et al. 2025). Given that insulin resistance, affecting 50%–70% of PCOS patients, has been shown to impair glycolysis in granulosa cells through downregulation of key glycolytic enzymes (Hu et al. 2024; Essah and Nestler 2007), GPT2 may mediate the effects of nutritional status and energy balance on ovarian function. In support of this notion, studies in metabolic tissues have demonstrated that GPT2 expression and activity are modulated by nutritional cues, including carbohydrate availability and amino acid supply (Baytas et al. 2022), and that its anaplerotic function is essential for sustaining TCA cycle flux and ATP production when glycolytic input is limited (Ponton‐Almodovar et al. 2025). This raises the possibility that dietary interventions, such as modulation of carbohydrate intake or bioactive nutrient supplementation, could influence glycolytic function in PCOS via pathways involving GPT2, warranting further investigation.

Despite these findings, several limitations should be acknowledged. First, this study was primarily based on two publicly available transcriptomic datasets with relatively limited sample sizes, which may not fully capture the biological and clinical heterogeneity of PCOS. Moreover, the integrated datasets were derived from different tissue sources, including granulosa cells and skeletal muscle, which may introduce tissue‐specific transcriptional variability. To mitigate this issue, differential expression analyzes were performed independently within each dataset, and only consistently dysregulated glycolysis‐related genes were retained for subsequent analyzes. Nevertheless, the identified molecular signatures should be interpreted as PCOS‐associated glycolytic alterations rather than tissue‐specific biomarkers. Furthermore, the relatively permissive differential expression criteria adopted in this exploratory study may have included genes with modest expression changes, and therefore the findings should be interpreted with appropriate caution. In addition, the molecular signatures and exploratory model were developed and evaluated within the same data framework and therefore require validation in independent clinical cohorts before broader application. Second, except for the functional characterization of GPT2 in KGN granulosa cells, the identified glycolysis‐associated genes were not further validated in clinical specimens or animal models. Likewise, immune infiltration analyzes were inferred computationally and warrant confirmation using experimental approaches such as flow cytometry or immunohistochemistry. Finally, although the KGN cell model provides a useful platform for investigating granulosa cell metabolism, it cannot fully recapitulate the complex metabolic and endocrine microenvironment of PCOS in vivo. Future studies integrating homogeneous tissue‐specific cohorts, large clinical populations, animal models, and mechanistic investigations will be essential to validate the biological functions of glycolysis‐associated regulators, clarify their tissue‐specific and systemic metabolic roles, and further evaluate their relevance to metabolic dysregulation and potential nutrition‐oriented interventions in PCOS.

## 4 Materials and Methods

### 4.1 Data Download

The expression profile data of polycystic ovary syndrome (PCOS) patients were downloaded from the GEO database (https://www.ncbi.nlm.nih.gov/geo/) (Barrett et al. 2013) via the R package GEOquery (Davis and Meltzer 2007), specifically from datasets GSE34526 (Kaur et al. 2012) and GSE6798 (Skov et al. 2007). The samples of datasets GSE34526 and GSE6798 are all from *Homo sapiens* . The samples are sourced from polycystic ovary syndrome cases, and the chip platform is GPL570. Refer to Table S8 for specific information. Among these, dataset GSE34526 comprises 7 PCOS patient samples and 3 matching normal tissue samples, while dataset GSE6798 contains 16 PCOS patient samples and 13 matching normal samples. Specifically, GSE34526 comprises samples derived from granulosa cells of ovarian follicles in PCOS patients and healthy controls, whereas GSE6798 includes data obtained from skeletal muscle tissues. The original study was primarily designed to characterize PCOS‐associated differential gene expression rather than to conduct experiments targeting glycolysis‐related metabolic interventions. All samples in the two GEO datasets were included in this study. The R package sva (Leek et al. 2012) was employed to perform batch effect removal on datasets GSE34526 and GSE6798. The R package limma (Ritchie et al. 2015) was utilized to standardize datasets GSE34526 and GSE6798, as well as to standardize and normalize the annotation probes.

Additionally, we retrieved Glycolysis Related Genes (GRGs) via the GeneCards database (Stelzer et al. 2016) The GeneCards database offers comprehensive data regarding human genes. We employed the term “Glycolysis” as the search query and retained only Glycolysis‐related genes (GRGs) that met the criteria of “Protein Coding” and “Relevance Score > 1”. In total, 1063 Glycolysis‐related genes (GRGs) were acquired. For detailed particulars, refer to Table S9.

### 4.2 PCOS‐Related Glycolysis‐Related Differentially Expressed Genes

The samples were divided into the Normal group and the PCOS group respectively. The R package “limma” was employed to conduct differential analysis of genes between the PCOS group and the Normal group. Given the distinct tissue origins of the two datasets (granulosa cells vs. skeletal muscle), differential expression analyzes were performed separately within each dataset to minimize the confounding effects of tissue‐specific transcriptional variation.

The initial screening thresholds for differentially expressed genes (DEGs) were set as | logFC | > 0 and nominal *p* value < 0.05. Among these, genes with logFC > 0 and nominal *p* value < 0.05 were defined as up‐regulated genes, and genes with logFC < 0 and nominal *p* value < 0.05 were defined as down‐regulated genes. The Benjamini‐Hochberg (BH) method was applied to control the false discovery rate, and the resulting adjusted *p*‐values were calculated for reference. Importantly, the relatively permissive nominal *p*‐value threshold was intentionally adopted at this initial screening stage to maximize sensitivity and capture a broad spectrum of PCOS‐associated transcriptional changes, thereby avoiding the potential loss of biologically relevant but modestly expressed genes. The robustness of candidate genes identified from this sensitive screening was subsequently reinforced through multiple machine‐learning algorithms (SVM, random forest, and LASSO regression) in the downstream analytical pipeline (see section 4.5).

Subsequent analyzes were restricted to glycolysis‐related genes that were consistently differentially expressed across both datasets. The results of the differential analysis were utilized to generate a volcano plot using the R package “ggplot2”.

To acquire Glycolysis‐Related Differentially Expressed Genes (GRDEGs) associated with PCOS, the intersections of all DEGs obtained from the two datasets and glycolysis‐related genes (GRGs) were determined. Specifically, glycolysis‐related genes (GRGs) were retrieved from the GeneCards database using the search term “Glycolysis” with criteria of “Protein Coding” and “Relevance Score > 1”, yielding a total of 1063 GRGs. A Venn diagram was drawn to obtain GRDEGs, and a heatmap was generated via the R package “pheatmap”.

### 4.3 Gene Ontology (GO) Enrichment Analysis

Gene Ontology (GO) analysis (Mi et al. 2019) is a prevalent method for large‐scale functional enrichment research, which encompasses Biological Process (BP), Cell Component (CC), and Molecular Function (MF). Gene Ontology (GO) enrichment analysis was performed on glycolysis—related differentially expressed genes (GRDEGs) using the R package “clusterProfiler” (Yu et al. 2012). The criteria for entry screening were that *p* adj < 0.05 and the FDR value (*q* value) < 0.05 were regarded as statistically significant. The *p* value correction method was Benjamini‐Hochberg (BH).

### 4.4 GSEA Enrichment Analysis

GSEA (Gene Set Enrichment Analysis) (Subramanian et al. 2005), also known as gene set enrichment analysis, is employed to assess the distribution tendency of genes within a predefined gene set in a gene table sorted by phenotype correlation, thereby determining its contribution to the phenotype. In this research, the genes in the datasets GSE34526 and GSE6798 were sorted according to logFC values. Subsequently, the R package clusterProfiler was utilized to conduct GSEA on all genes in the datasets GSE34526 and GSE6798. The parameters employed in the GSEA are as follows: the seed is 2020, the number of calculations is 1000, the minimum number of genes contained in each gene set is 10, and the maximum number of genes is 500. The gene set c2.cp.all.v2022.1.Hs.symbols.gmt [All Canonical Pathways] (3050) was obtained through the Molecular Signatures Database (MSigDB) database (Molecular signatures database 3.0) (Liberzon et al. 2011), and GSEA was performed. The screening criteria for GSEA are *p* adj < 0.05 and FDR value (*q* value) < 0.05, and the *p*‐value correction method is Benjamini‐Hochberg (BH).

### 4.5 Exploratory Classification and Candidate Molecular‐Signature Analysis

To prioritize glycolysis‐related genes with potential classification relevance, support vector machine (SVM), random forest (RF), logistic regression, and least absolute shrinkage and selection operator (LASSO) regression analyzes were performed using the GRDEGs in the GSE6798 dataset. First, an SVM algorithm was applied, and candidate features were selected according to classification accuracy and error rate. Random forest analysis was subsequently performed using the randomForest package with set.seed (250) and ntree = 1000, and candidate genes were ranked according to IncNodePurity.

Logistic regression analysis was performed to evaluate the association between individual GRDEGs and sample classification (PCOS versus control), with *p* value < 0.05 used as the screening criterion. Genes retained by logistic regression, SVM, and random forest analyzes were intersected to identify common candidate genes. LASSO regression was then performed on these candidates using the glmnet package with set.seed (500) and family = “binomial” to further refine the candidate molecular signature. The genes retained by LASSO were defined as hub genes for subsequent analyzes.

A classification score was calculated for each sample based on the expression levels of the retained hub genes and their corresponding LASSO coefficients. Samples were ranked according to the classification score and divided into high‐ and low‐score groups using the median value for subsequent exploratory analyzes.

A nomogram was constructed using the rms package to visualize the relative contributions of the hub genes to the candidate molecular signature. Calibration analysis was performed to examine the agreement between predicted and observed sample classifications within the analyzed dataset. Decision curve analysis (DCA) was additionally used as an exploratory assessment of the classification performance of the candidate molecular signature across different threshold probabilities.

Receiver operating characteristic (ROC) curve analysis was performed to characterize the discriminatory performance of individual hub genes in distinguishing PCOS from control samples within GSE6798. The area under the ROC curve (AUC) was calculated as a descriptive measure of classification performance. These analyzes were considered exploratory and were not intended to establish clinical diagnostic performance.

### 4.6 Gene Set Variation Analysis (GSVA)

GSVA (Gene Set Variation Analysis) (Hanzelmann et al. 2013) namely gene set variation analysis, is a non—parametric unsupervised analysis method. Primarily, it transforms the expression matrix of genes among different samples into the expression of gene sets among samples. The quantitative matrix is utilized to assess the gene set enrichment results of the chip nuclear transcriptome. This assessment serves to determine whether different pathways are enriched among different samples. The h.all.v7.4.symbols.gmt gene set is obtained from the Molecular Signatures Database (MSigDB), and gene set variation analysis (GSVA) is conducted on all genes of the data set GSE6798 to compare functional enrichment between the high‐ and low‐score groups defined by the median classification score of the candidate molecular signature. The screening criterion for gene set variation analysis (GSVA) is *p* value < 0.05.

### 4.7 Protein–Protein Interaction (PPI) Network

The PPI Network (Protein—protein Interaction Network), also known as the protein—protein interaction network, is composed of mutually interacting proteins. It participates in various aspects of the life process, such as biological signal transmission, gene expression regulation, energy and material metabolism, and cell cycle regulation. Conducting a systematic analysis of the interactions between proteins in biological systems holds significant importance for comprehending the working principles of proteins within biological systems, understanding the reaction mechanisms of biological signals and energy—substance metabolism under special physiological states like diseases, and discerning the functional connections between proteins.

The GeneMANIA (Franz et al. 2018) database (http://genemania.org) is a website designed for the analysis and prediction of gene functions in an input gene list to determine and generate gene priorities. The GeneMANIA database was utilized to construct a protein—protein interaction network from hub genes.

Proteins are crucial for life, and understanding their structure can contribute to the understanding of their corresponding functions. The Alphafold (https://www.alphafold.ebi.ac.uk/) website (Varadi et al. 2022) first proposed that protein structure can be predicted with atomic precision using computational methods without a homologous template. The predicted structures encompass 98.5% of known human proteins and an equivalent proportion of proteins from other organisms. The Alphafold2 website was employed to predict the protein structures of hub genes in the PPI network and present the results.

### 4.8 Construct the mRNA‐miRNA, mRNA‐TF, and mRNA‐RBP Interaction Network

MicroRNA (miRNA) exerts a significant regulatory function during the processes of biological development and evolution. It can regulate a diverse array of target genes, and conversely, a single target gene can be regulated by multiple miRNAs. To examine the relationship between hub genes and miRNAs, miRNAs associated with hub genes were retrieved from the miRDB (Chen and Wang 2020) database. Subsequently, the mRNA—miRNA regulatory network was visualized using Cytoscape (version 3.9.1) (Shannon et al. 2003) software.

Transcriptional factors (TFs) govern gene expression through interactions with hub genes at the post—transcriptional stage. Key transcription factors were extracted from the ChIPBase database (Zhou et al. 2017) and their regulatory impacts on hub genes were analyzed. The mRNA—TF regulatory network was then constructed using Cytoscape software.

RNA‐binding protein (RBP) (Singh 2021) assumes a crucial role in the gene regulation process and is essential for the regulation of vital biological activities, such as RNA synthesis, alternative splicing, modification, transport, and translation. Based on the StarBase v3.0 database (Li et al. 2014), the target RNA—binding proteins (RBPs) of the hub gene were predicted, and the mRNA—RBP regulatory network was visualized using Cytoscape software.

### 4.9 Immune Infiltration Analysis

ssGSEA (Single‐Sample Gene‐Set Enrichment Analysis) (Xiao et al. 2020) denotes single‐sample gene set enrichment analysis, quantifying the relative abundance of each immune cell infiltrate. Initially, specific infiltrating immune cell types are identified, including Activated CD8 T cells, Activated dendritic cells, Gamma delta T cells, Natural killer cells, Regulatory T cells, and other human immune cell subtypes. Subsequently, the enrichment score calculated by ssGSEA analysis represents the relative abundance of each immune cell infiltration within every sample. Samples exhibiting a *p* value < 0.05 are then filtered, yielding the final immune cell infiltration matrix. We visually contrasted immune cell infiltration differences between the PCOS and Normal groups in the GSE6798 dataset using group comparison charts. Furthermore, employing the R package pheatmap, we generated correlation heatmaps to illustrate correlations between immune cells themselves, as well as between hub genes and immune cells within the GSE6798 dataset.

CIBERSORT (Newman et al. 2015) deconvolutes the transcriptome expression matrix using linear support vector regression to estimate immune cell composition and abundance within mixed populations. Employing the CIBERSORT algorithm combined with the LM22 signature gene matrix, data exhibiting immune cell enrichment scores greater than zero are filtered, yielding the specific immune cell infiltration matrix results. The R package ggplot2 then generated a proportion histogram visualizing the expression differences of LM22 immune cells between the PCOS group and the Normal group in dataset GSE6798. Concurrently, the R package pheatmap produced a correlation heatmap depicting associations among LM22 immune cells themselves and between hub genes and LM22 immune cells.

### 4.10 Cell Culture and Functional Assays

The human granulosa cell line KGN was sourced from the Cell Bank of the Chinese Academy of Sciences (Shanghai, China) and cultured in a complete medium of DMEM/F12 supplemented with 10% fetal bovine serum (FBS) under standard conditions, utilizing a Thermo Scientific incubator and centrifuge. As a preliminary platform for cellular functional validation in this study, the KGN cell model was primarily used to analyze the functional roles of candidate molecules such as GPT2, rather than to directly recapitulate the pathological status of polycystic ovary syndrome (PCOS) patients in vivo. All cell experimental results are provided solely as references for mechanistic exploration and cannot fully substitute for conclusions derived from clinical samples or animal models. Future studies will incorporate more physiologically relevant research systems to complement and refine these findings.

All procedures—including cell thawing, passaging, and cryopreservation—were meticulously performed under strict sterile protocols to guarantee optimal cell viability and experimental reliability. Furthermore, for comprehensive molecular and biochemical analyzes, quantitative real‐time polymerase chain reaction (RT‐PCR) was employed to precisely quantify mRNA levels of GPT2, CYP19A1, and key glycolytic genes (HK2, PKM2, LDHA, PFKP). Changes in GPT2 and associated protein expression were assessed via Western blotting. Enzyme‐linked immunosorbent assay (ELISA) was utilized to measure testosterone and estradiol (E2) secretion. Cellular ATP and lactate levels were determined using an enhanced ATP assay kit and the Amplex Red L‐lactate assay kit, respectively. Cells were allocated into four experimental groups: a control group maintained under routine culture conditions; a negative control siRNA plus exogenous androgen group (si‐ctrl+FSK + A), in which cells were transfected with control siRNA followed by treatment with Forskolin (1 μM) and Testosterone (100 nM); a GPT2‐specific siRNA plus exogenous androgen group (si‐GPT2 + FSK + A), in which cells received GPT2‐targeted siRNA prior to the same hormonal stimulation; and a GPT2‐specific siRNA plus glycolysis agonist plus exogenous androgen group (si‐GPT2 + DPI + FSK + A), in which the glycolysis agonist DPI (500 nM) was added after GPT2 knockdown (Dong et al. 2024). The GPT2 siRNA sequences were Sense 5′‐GGGAAGAGAAGCUCUUUCUTT‐3′ and Antisense 5′‐AGAAAGAGCUUCUCUUCCCTT‐3′. Transfections were performed using Lipofectamine 3000, achieving a knockdown efficiency of at least 70%. The RT‐PCR primer information for target genes and antibodies Informations were listed in Tables 1 and 2.

**TABLE 1:** Sequences of qPCR primers for target genes.

Name	Sequence	
GPT2	F	GTGATGGCACTATGCACCTAC
	R	TTCACGGATGCAGTTGACACC
HK2	F	GAGCCACCACTCACCCTACT
	R	CCAGGCATTCGGCAATGTG
PKM2	F	ATGTCGAAGCCCCATAGTGAA
	R	TGGGTGGTGAATCAATGTCCA
LDHA	F	ATGGCAACTCTAAAGGATCAGC
	R	CCAACCCCAACAACTGTAATCT
PFKP	F	GCATGGGTATCTACGTGGGG
	R	CTCTGCGATGTTTGAGCCTC
CYP19A1	F	TGGAAATGCTGAACCCGATAC
	R	AATTCCCATGCAGTAGCCAGG

**TABLE 2:** Reagents and antibodies information.

Name	Company	Number	Dilution ratio
GPT2	Wuhan Sanying Biotechnology Co. Ltd.	16,757‐1‐ap	1:1000
HK2	Hua'an Biotechnology Co. Ltd.	HA722933	1:3000
PKM2	Hua'an Biotechnology Co. Ltd.	HA723370	1:10000
PFKP	MCE	HY‐P81854	1:1000
CYP19A1	MCE	HY‐P80547	1:1000
Vinculin	ABclonal	A27905	1:2000
Goat Anti‐Rabbit	Wuhan Sanying Biotechnology Co. Ltd.	SA00001‐2	1:4000
Goat Anti‐Mouse	Wuhan Sanying Biotechnology Co. Ltd.	SA00001‐1	1:4000

### 4.11 Statistical Analysis

All data processing and analysis were conducted using R software (Version 4.2.2). Unless otherwise noted, the normality of continuous variables was evaluated using the Shapiro–Wilk test. For comparisons between two groups (e.g., PCOS vs. Normal), independent‐samples Student's *t*‐tests were applied when data met normality and homogeneity of variance; otherwise, the Mann–Whitney *U* test (Wilcoxon rank‐sum test) was used. For comparisons among three or more groups (e.g., PCOS subtypes), one‐way ANOVA with appropriate post hoc tests was performed when parametric assumptions were satisfied; otherwise, the Kruskal–Wallis test followed by post hoc pairwise comparisons was applied. Correlations between molecular markers were assessed using Spearman's correlation analysis. All statistical tests were two‐tailed unless stated otherwise, and *p*‐values < 0.05 were considered statistically significant.

## 5 Conclusions

This study comprehensively characterized glycolytic dysregulation in PCOS and identified candidate glycolysis‐related genes associated with immune‐metabolic remodeling. Functional validation further supports GPT2 as a potential regulator of granulosa cell glycolytic metabolism and metabolic homeostasis. These findings may provide new insights into the metabolic mechanisms underlying PCOS and may offer a molecular basis for future studies investigating nutrition‐related metabolic regulation in PCOS.

## Author Contributions

**Meili Xi:** conceptualization, methodology, software, investigation, formal analysis, supervision, writing – review and editing, writing – original draft, visualization, resources, data curation. **Rongkui Luo:** conceptualization, methodology, software, writing – original draft, investigation, validation, formal analysis. **Jiarong Zhang:** methodology, supervision, resources. **Weihong Huang:** data curation, software. **Li Ma:** investigation, conceptualization, validation. **Yunyan Sun:** writing – original draft, writing – review and editing. **Aimin Ren:** writing – review and editing, writing – original draft, project administration.

## Funding

This work was supported by National Natural Science Foundation of China (Grant 81971345).

## Ethics Statement

The study was approved by the Ethics Committee of Zhongshan Hospital, Fudan University (B2020‐115R2).

## Conflicts of Interest

The authors declare no conflicts of interest.

## Supporting information


**Figure S1:** Batch effect removal for datasets GSE34526 and GSE6798.
**Figure S2:** Gene set enrichment analysis (GSEA) of datasets GSE34526 and GSE6798.
**Table S1:** GO enrichment analysis results.
**Table S2:** GSVA enrichment analysis results of GSE6798.
**Table S3:** GSEA enrichment analysis results of GSE34526.
**Table S4:** GSEA enrichment analysis results of GSE6798.
**Table S5:** mRNA‐miRNA interaction network nodes.
**Table S6:** mRNA‐TF interaction network nodes.
**Table S7:** mRNA‐RBP interaction network nodes.
**Table S8:** PCOS data set information list.

**Table S9:** gly‐gene.

## Data Availability

The data presented in this study are available in the article and its Supporting Information. Further inquiries can be directed to the corresponding authors. The public datasets used for bioinformatics analysis in this study are available in the Gene Expression Omnibus (GEO) database: (1) Dataset GSE34526, with the access URL: https://www.ncbi.nlm.nih.gov/geo/query/acc.cgi?acc=GSE34526 (2) Dataset GSE6798, with the access URL https://www.ncbi.nlm.nih.gov/geo/query/acc.cgi?acc=GSE6798. Code Availability: The custom code used for data processing and analysis in this study is available from the corresponding author upon reasonable request.
