# Supplementary material for: Integrative Transcriptomic Analysis and Functional Validation Implicate GPT2 in Glycolytic Regulation in Polycystic Ovary Syndrome
Source: Food Sci Nutr. 2026 Sep 6;14(9):e72326. doi: 10.1002/fsn3.72326 (PMC13547747; doi:10.1002/fsn3.72326)
Supplement: Supplementary file 1 [file FSN3-14-e72326-s001.docx]

**Supplement**

**Supplementary Figure 1: Batch effect removal for datasets GSE34526 and GSE6798.**

*A. Distribution box plot of the data set G SE34526 before de-batch processing . B. Distribution box plot of the data set G SE34526 after de-batch processing. C. Distribution box plot of the data set G SE6798 before de-batch processing . D. Distribution box plot of the data set G SE6798 after de-batch processing . PCOS , Polycystic Ovary Syndrome .*

**Supplementary Figure 2: Gene set enrichment analysis (GSEA) of datasets GSE34526 and GSE6798.**

*A. The GSEA enrichment analysis of the data set GSE34526 mainly contains 4 main biological characteristics. BE. All genes in the data set GSE34526 are significantly enriched in LYSOSOME ( B ), HEMATOPOIETIC_CELL_LINEAGE ( C ), APOPTOSIS ( D ), PYROPTOSIS (E) and other pathways. F. The GSEA enrichment analysis of the data set GSE6798 mainly contains 4 main biological characteristics. GJ. All genes in the data set GSE34526 are significantly enriched in pathways such as MITOCHONDRIAL_TRANSLATION ( G ), FATTY_ACID_METABOLISM ( H ), NEUTROPHIL_DEGRANULATION ( I ), MIRNA_TARGETS_IN_ECM_AND_MEMBRANE_RECEPTORS (J). The significant enrichment screening criteria for GSEA enrichment analysis are p.adj < 0.05 and FDR value (q value) < 0.05 , and the p value correction method is Benjamini -Hochberg ( BH ) . PCOS , Polycystic Ovary Syndrome ; GSEA: Gene Set Enrichment Analysis.*

**Supplementary Table 1:** GO enrichment analysis results.

| Ontology | ID | Description | GeneRatio | BgRatio | p.adjust | q.value |
| --- | --- | --- | --- | --- | --- | --- |
| BP | GO:0006096 | glycolytic process | 3/12 | 81/18800 | 0.002042 | 0.001183 |
| BP | GO:0006757 | ATP generation from ADP | 3/12 | 82/18800 | 0.002042 | 0.001183 |
| BP | GO:0046939 | nucleotide phosphorylation | 3/12 | 100/18800 | 0.002042 | 0.001183 |
| BP | GO:0006090 | pyruvate metabolic process | 3/12 | 106/18800 | 0.002054 | 0.00119 |
| BP | GO:0009150 | purine ribonucleotide metabolic process | 4/12 | 366/18800 | 0.002834 | 0.001642 |
| MF | GO:0061629 | RNA polymerase II-specific DNA-binding transcription factor binding | 4/12 | 348/18410 | 0.003746 | 0.00174 |
| MF | GO:0140297 | DNA-binding transcription factor binding | 4/12 | 470/18410 | 0.005997 | 0.002785 |

GO：Gene Ontology.

BP：iological process.

MF：olecular function.

**Supplementary Table 2: GSVA enrichment analysis results of GSE6798.**

| Description | logFC | p.value |
| --- | --- | --- |
| REACTOME_BETA_OXIDATION_OF_LAUROYL_COA_TO_DECANOYL_COA_COA | -0.65255 | 0.001022 |
| TAKAYAMA_BOUND_BY_AR | -0.62998 | 4.40E-05 |
| REACTOME_THYROXINE_BIOSYNTHESIS | -0.59797 | 0.000409 |
| REACTOME_BETA_OXIDATION_OF_HEXANOYL_COA_TO_BUTANOYL_COA | -0.58446 | 0.01154 |
| REACTOME_BETA_OXIDATION_OF_OCTANOYL_COA_TO_HEXANOYL_COA | -0.56924 | 0.009118 |
| WP_KETONE_BODIES_SYNTHESIS_AND_DEGRADATION | -0.54549 | 0.003804 |
| REACTOME_REVERSIBLE_HYDRATION_OF_CARBON_DIOXIDE | -0.52068 | 0.004311 |
| HOFFMAN_CLOCK_TARGETS_DN | -0.50252 | 0.009907 |
| REACTOME_REGULATION_BY_C_FLIP | -0.49942 | 0.006797 |
| REACTOME_KETONE_BODY_METABOLISM | -0.47719 | 0.002024 |
| IBRAHIM_NRF3_UP | 0.491572 | 0.041975 |
| WP_ETHANOL_METABOLISM_PRODUCTION_OF_ROS_BY_CYP2E1 | 0.491786 | 0.005955 |
| REACTOME_SLBP_DEPENDENT_PROCESSING_OF_REPLICATION_DEPENDENT_HISTONE_PRE_MRNAS | 0.520394 | 0.003941 |
| TURJANSKI_MAPK8_AND_MAPK9_TARGETS | 0.533779 | 0.002779 |
| ZHENG_RESPONSE_TO_ARSENITE_UP | 0.534325 | 0.008945 |
| CALVET_IRINOTECAN_SENSITIVE_VS_REVERTED_DN | 0.539265 | 0.020631 |
| WP_DDX1_AS_A_REGULATORY_COMPONENT_OF_THE_DROSHA_MICROPROCESSOR | 0.554215 | 0.026554 |
| REACTOME_METALLOTHIONEINS_BIND_METALS | 0.609692 | 0.012987 |
| REACTOME_ACTIVATED_PKN1_STIMULATES_TRANSCRIPTION_OF_AR_ANDROGEN_RECEPTOR_REGULATED_GENES_KLK2_AND_KLK3 | 0.628302 | 0.000673 |
| REACTOME_ACTIVATION_OF_NOXA_AND_TRANSLOCATION_TO_MITOCHONDRIA | 0.647375 | 0.000771 |

**Supplementary Table 3: GSEA enrichment analysis results of GSE34526.**

| Description | setSize | NES | p.adjust | qvalues |
| --- | --- | --- | --- | --- |
| REACTOME_PYROPTOSIS | 24 | 1.734612 | 0.040142 | 0.031891 |
| WP_APOPTOSIS | 82 | 1.881611 | 0.002108 | 0.001675 |
| KEGG_HEMATOPOIETIC_CELL_LINEAGE | 83 | 2.306966 | 7.53E-07 | 5.98E-07 |
| KEGG_LYSOSOME | 116 | 2.341889 | 5.32E-08 | 4.23E-08 |
| NABA_CORE_MATRISOME | 255 | -1.37834 | 0.046722 | 0.037119 |
| WP_ENDODERM_DIFFERENTIATION | 136 | -1.51782 | 0.031232 | 0.024813 |
| REACTOME_CILIUM_ASSEMBLY | 183 | -1.54011 | 0.006776 | 0.005383 |
| KEGG_BASAL_CELL_CARCINOMA | 52 | -1.67376 | 0.034234 | 0.027198 |
| WP_BARDETBIEDL_SYNDROME | 78 | -1.69996 | 0.024294 | 0.0193 |
| REACTOME_NETRIN_1_SIGNALING | 49 | -1.7296 | 0.028537 | 0.022671 |
| REACTOME_INTRAFLAGELLAR_TRANSPORT | 46 | -1.74133 | 0.025558 | 0.020304 |
| WP_CILIOPATHIES | 157 | -1.74433 | 0.000525 | 0.000417 |
| KEGG_LEISHMANIA_INFECTION | 67 | 2.642299 | 2.48E-08 | 1.97E-08 |
| WP_COMPLEMENT_SYSTEM | 91 | 2.587906 | 2.48E-08 | 1.97E-08 |
| KEGG_ASTHMA | 26 | 2.371085 | 4.34E-06 | 3.44E-06 |

GEO：Gene Expression Omnibus.

PCOS：Polycystic ovary Syndrome

GSEA：Gene Set Enrichment Analysis.

### Supplementary Table 4: GSEA enrichment analysis results of GSE6798.

| Description | setSize | NES | p.adjust | qvalues |
| --- | --- | --- | --- | --- |
| WP_MIRNA_TARGETS_IN_ECM_AND_MEMBRANE_RECEPTORS | 21 | 1.904076 | 0.032166 | 0.030813 |
| REACTOME_NEUTROPHIL_DEGRANULATION | 448 | 1.630993 | 0.007028 | 0.006732 |
| REACTOME_FATTY_ACID_METABOLISM | 159 | -1.65625 | 0.032166 | 0.030813 |
| REACTOME_MITOCHONDRIAL_TRANSLATION | 91 | -1.98201 | 0.008378 | 0.008025 |
| WP_PATHOGENIC_ESCHERICHIA_COLI_INFECTION | 50 | 2.107889 | 0.008378 | 0.008025 |
| PID_TOLL_ENDOGENOUS_PATHWAY | 23 | 2.08573 | 0.010667 | 0.010218 |
| KEGG_PATHOGENIC_ESCHERICHIA_COLI_INFECTION | 51 | 2.063928 | 0.008378 | 0.008025 |
| REACTOME_STRIATED_MUSCLE_CONTRACTION | 35 | 2.049191 | 0.019319 | 0.018506 |
| WP_STRIATED_MUSCLE_CONTRACTION_PATHWAY | 37 | 1.970596 | 0.033249 | 0.031851 |
| WP_INCLUSION_BODY_MYOSITIS | 11 | 1.96561 | 0.046183 | 0.044241 |
| WP_TLR4_SIGNALING_AND_TOLERANCE | 28 | 1.91096 | 0.032166 | 0.030813 |
| REACTOME_RESPIRATORY_ELECTRON_TRANSPORT | 93 | -1.88471 | 0.009009 | 0.00863 |
| REACTOME_RESPONSE_TO_METAL_IONS | 12 | -1.98082 | 0.033249 | 0.031851 |
| REACTOME_SIGNALING_BY_PDGF | 58 | 1.81345 | 0.043341 | 0.041518 |
| REACTOME_MUSCLE_CONTRACTION | 195 | 1.779579 | 0.008378 | 0.008025 |

GEO：Gene Expression Omnibus.

PCOS：Polycystic ovary Syndrome

GSEA：Gene Set Enrichment Analysis.

### Supplementary Table 5: mRNA-miRNA interaction network nodes.

| miRNA |  | mRNA | miRNA |  | mRNA |
| --- | --- | --- | --- | --- | --- |
| hsa-miR-8485 | - | MLXIPL | hsa-miR-548ah-5p | - | AMPD3 |
| hsa-miR-5590-5p | - | C5AR1 | hsa-miR-27a-3p | - | AMPD3 |
| hsa-miR-4695-3p | - | C5AR1 | hsa-miR-9985 | - | AMPD3 |
| hsa-miR-153-5p | - | C5AR1 | hsa-miR-616-5p | - | C5AR1 |
| hsa-miR-765 | - | C5AR1 | hsa-miR-670-3p | - | C5AR1 |
| hsa-miR-6778-3p | - | C5AR1 | hsa-miR-373-5p | - | C5AR1 |
| hsa-miR-513a-5p | - | AMPD3 | hsa-miR-371b-5p | - | C5AR1 |
| hsa-miR-6885-3p | - | MLXIPL | hsa-miR-664a-3p | - | C5AR1 |
| hsa-miR-7106-5p | - | PDLIM7 | hsa-miR-3689d | - | MLXIPL |
| hsa-miR-6851-5p | - | PDLIM7 | hsa-miR-6851-5p | - | MLXIPL |
| hsa-miR-8485 | - | AMPD3 | hsa-miR-6769a-3p | - | AMPD3 |
| hsa-miR-3662 | - | AMPD3 | hsa-miR-4524b-3p | - | AMPD3 |
| hsa-miR-27b-3p | - | AMPD3 | hsa-miR-877-3p | - | AMPD3 |
| hsa-miR-3609 | - | AMPD3 | hsa-miR-11181-3p | - | C5AR1 |

“miRNA”and“mRNA”represent node；“-”represent edge.

### Supplementary Table 6: mRNA-TF interaction network nodes.

| TF |  | mRNA | TF |  | mRNA |
| --- | --- | --- | --- | --- | --- |
| CTCF | - | PDLIM7 | EGR1 | - | PDLIM7 |
| CTCF | - | MLXIPL | SPI1 | - | MLXIPL |
| CTCF | - | AMPD3 | TEAD4 | - | MLXIPL |
| CEBPB | - | PDLIM7 | SRF | - | PDLIM7 |
| RAD21 | - | PDLIM7 | MYC | - | MLXIPL |
| SPI1 | - | PDLIM7 | USF1 | - | MLXIPL |
| MAX | - | MLXIPL | EP300 | - | PDLIM7 |
| FOSL2 | - | MLXIPL | ERG | - | PDLIM7 |
| RAD21 | - | MLXIPL | REST | - | MLXIPL |
| NRF1 | - | PDLIM7 | ELF1 | - | PDLIM7 |
| FOS | - | MLXIPL | FOS | - | PDLIM7 |
| JUN | - | MLXIPL | GATA2 | - | PDLIM7 |
| JUND | - | MLXIPL | MAZ | - | PDLIM7 |
| CEBPA | - | PDLIM7 | MYC | - | PDLIM7 |
| STAG1 | - | PDLIM7 | STAT3 | - | PDLIM7 |
| SMC3 | - | PDLIM7 | TBP | - | PDLIM7 |
| TAL1 | - | PDLIM7 | TCF12 | - | PDLIM7 |

“TF”and“mRNA”represent node；“-”represent edge；

TF：Transcription factors.

**Supplementary Table 7: mRNA-RBP interaction network nodes.**

| RBP |  | mRNA |  | RBP |  | mRNA |
| --- | --- | --- | --- | --- | --- | --- |
| PABPN1 | - | MLXIPL |  | SRSF1 | - | PDLIM7 |
| MSI2 | - | PDLIM7 |  | TIA1 | - | PDLIM7 |
| AQR | - | AMPD3 |  | YTHDC1 | - | PDLIM7 |
| DAP3 | - | PDLIM7 |  | EIF3B | - | AMPD3 |
| U2AF2 | - | PDLIM7 |  | RBM10 | - | MLXIPL |
| YTHDF2 | - | PDLIM7 |  | ALYREF | - | PDLIM7 |
| ALYREF | - | AMPD3 |  | CPSF1 | - | PDLIM7 |
| CELF2 | - | AMPD3 |  | CTCF | - | PDLIM7 |
| AQR | - | PDLIM7 |  | FAM120A | - | PDLIM7 |
| DDX21 | - | PDLIM7 |  | RBFOX2 | - | PDLIM7 |
| DDX3X | - | PDLIM7 |  | SCAF8 | - | PDLIM7 |
| PTBP1 | - | PDLIM7 |  | TRMT10A | - | PDLIM7 |
| SF3B4 | - | PDLIM7 |  | YTHDF3 | - | PDLIM7 |

“RBP”and“mRNA”represent node；“-”represent edge.

RBP：RNA binding protein.

**Supplementary Table 8: PCOS data set information list.**

|  | **GSE34526** | **GSE6798** |
| --- | --- | --- |
| Platform | GPL570 | GPL570 |
| Species | Homo sapiens | Homo sapiens |
| Tissue | granulosa cells | skeletal muscle |
| Samples in PCOS group | 7 | 16 |
| Samples in Normal group | 3 | 13 |
| Reference | Differential gene expression in granulosa cells from polycystic ovary syndrome patients with and without insulin resistance: identification of susceptibility gene sets through network anaalysis. | Reduced expression of nuclear-encoded genes involved in mitochondrial oxidative metabolism in skeletal muscle of insulin-resistant women with polycystic ovary syndrome. |

GEO：Gene Expression Omnibus.

PCOS：Polycystic ovary Syndrome.

**Supplementary Table 9: gly-gene.csv**
